# Neighboring inteins interfere with one another's homing capacity

**DOI:** 10.1093/pnasnexus/pgad354

**Published:** 2023-10-27

**Authors:** Israela Turgeman-Grott, Danielle Arsenault, Dekel Yahav, Yutian Feng, Guy Miezner, Doron Naki, Omri Peri, R Thane Papke, Johann Peter Gogarten, Uri Gophna

**Affiliations:** The Shmunis School of Biomedicine and Cancer Research, Faculty of Life Sciences, Tel Aviv University, P.O. Box 39040, 6997801 Tel Aviv, Israel; Department of Molecular and Cell Biology, University of Connecticut, 91 North Eagleville Road, Storrs, CT 06268-3125, USA; The Shmunis School of Biomedicine and Cancer Research, Faculty of Life Sciences, Tel Aviv University, P.O. Box 39040, 6997801 Tel Aviv, Israel; Department of Molecular and Cell Biology, University of Connecticut, 91 North Eagleville Road, Storrs, CT 06268-3125, USA; The Shmunis School of Biomedicine and Cancer Research, Faculty of Life Sciences, Tel Aviv University, P.O. Box 39040, 6997801 Tel Aviv, Israel; The Shmunis School of Biomedicine and Cancer Research, Faculty of Life Sciences, Tel Aviv University, P.O. Box 39040, 6997801 Tel Aviv, Israel; The Shmunis School of Biomedicine and Cancer Research, Faculty of Life Sciences, Tel Aviv University, P.O. Box 39040, 6997801 Tel Aviv, Israel; Department of Molecular and Cell Biology, University of Connecticut, 91 North Eagleville Road, Storrs, CT 06268-3125, USA; Department of Molecular and Cell Biology, University of Connecticut, 91 North Eagleville Road, Storrs, CT 06268-3125, USA; Institute for Systems Genomics, University of Connecticut, 67 North Eagleville Road, Storrs, CT 06268-3003, USA; The Shmunis School of Biomedicine and Cancer Research, Faculty of Life Sciences, Tel Aviv University, P.O. Box 39040, 6997801 Tel Aviv, Israel

**Keywords:** intein, homing endonuclease, haloarchaea, co-homing

## Abstract

Inteins are mobile genetic elements that invade conserved genes across all domains of life and viruses. In some instances, a single gene will have several intein insertion sites. In Haloarchaea, the minichromosome maintenance (MCM) protein at the core of replicative DNA helicase contains four intein insertion sites within close proximity, where two of these sites (MCM-a and MCM-d) are more likely to be invaded. A haloarchaeon that harbors both MCM-a and MCM-d inteins, *Haloferax mediterranei*, was studied in vivo to determine intein invasion dynamics and the interactions between neighboring inteins. Additionally, invasion frequencies and the conservation of insertion site sequences in 129 Haloferacales *mcm* homologs were analyzed to assess intein distribution across the order. We show that the inteins at MCM-a and MCM-d recognize and cleave their respective target sites and, in the event that only one empty intein invasion site is present, readily initiate homing (i.e. single homing). However, when two inteins are present co-homing into an intein-free target sequence is much less effective. The two inteins are more effective when invading alleles that already contain an intein at one of the two sites. Our in vivo and computational studies also support that having a proline in place of a serine as the first C-terminal extein residue of the MCM-d insertion site prevents successful intein splicing, but does not stop recognition of the insertion site by the intein's homing endonuclease.

**Significance Statement**

Inteins are molecular parasites residing within important protein-coding genes. They can infect intein-free genes when they come into contact with them via sex or horizontal gene transfer. In many archaea, two inteins reside within the same gene, yet the relationships between them have not been explored. We now show that two natural inteins, present in an archaeon, have retained full independent invasion capacity, and each of them recognizes a distinct part of the gene. Surprisingly, when both inteins try to invade different sites at the same time this results in very low invasion rates. This implies that double inteins mostly infect single intein versions of the gene, which are common in the genomes of archaeal species that have double inteins.

## Introduction

Inteins are mobile genetic elements that invade conserved genes across microbes from all domains of life and their viruses, including chloroplasts of uni- and multicellular algae. Inteins splice themselves out of host proteins post-translationally (for reviews see (1–4)). Most known inteins also contain a homing endonuclease (HEN) domain that makes a double-strand break in the target allele—the version of the gene that does not contain an intein at a highly specific sequence, known as the homing site. When this break is repaired via the homologous recombination machinery, the template is the intein-containing allele, which promotes intein invasion by gene conversion. The HENs in the inteins investigated here belong the LAGLIDADG/DOD familiy, which is named after the amino acids present in a conserved sequence motif (5). The relationship between the two functionally independent domains of the intein, the self-splicing domain and the HEN can be described as mutualistic: the self-splicing domain (present in Hedgehog proteins and INTeins, sometimes called HINT domain) minimizes the detrimental effect on the host cell and host protein (called the extein), while the HEN domain provides transmissibility. The splicing domain cannot degenerate without impacting host fitness, since inteins tend to reside in genes that are essential or at least highly important to the host cell (6, 7). In contrast, the HEN domain can undergo functional degeneration, as has been demonstrated in yeast (8). Thus, when transmissibility is not a strong selective factor (i.e. the intein has become fixed in the population), the HEN domain may accumulate mutations and degenerate (8–10). While many inteins have lost their HEN domain, so called mini-inteins (11), in vitro studies have shown that in some cases, protein splicing depends on the existence of the HEN domain (12), or on a region separating the two parts of the splicing domain that provide structural flexibility required for splicing (13).

Many genes, especially in euryarchaea, have more than one potential intein insertion site, and often two or more of these sites are occupied simultaneously. As an example, the *polB* gene encoding the replicative DNA polymerase in halophilic archaea nearly always contains an intein at one of three potential insertion sites; the *Haloquadratum walsbyi polB* homolog harbors three large inteins that still contain HEN domains in all three sites (14). In most of these cases, the inteins contain a putative HEN domain, judging by their length and presence of HEN-associated motifs (Table 1). Inactivated intron-encoded HENs in bacteriophages have been shown to be mobilized by active neighboring HENs since the gene conversion event can involve substantial flanking DNA regions beyond the homing site (15, 16). This raised the question, are all HEN domains in a multi-intein Open Reading Frame (ORF) still fully functional, or does one of them hitch-hike and “co-home” and is thus free to degenerate?

**Table 1.** Multiple intein containing genes in archaea.

Group (total genomes)^a^	Multi-intein genes	% of multi-inteins with detectable HEN^b^	Single-intein genes	% of single inteins with detectable HEN
Archaeoglobales (8)	1	100% (2)	13	100%
Class II Methanogens (36)	0	n/a	12	25.0%
Diaforarchaea (15)	2	0% (4)	5	40.0%
Halobacteriales (66)	41	57.9% (95)	162	59.3%
Thermococcales (41)	47	72.4% (105)	172	83.7%
Methanomada (36)	5	63.6% (11)	70	62.9%
Crenarchaeota (56)	0	n/a	10	30.0%
Korarchaeota (1)	0	n/a	0	n/a
Thaumarchaeota (18)	0	n/a	1	0%
DPANN (2)	0	n/a	1	100%
Asgard (1)	0	n/a	0	n/a
Unclassified (1)	0	n/a	5	80.0%

^a^The number in the parentheses gives the total number of genomes analyzed for each group.

^b^The number in the parentheses indicates the total number of inteins in multiple intein containing genes.

**Fig. 1.**
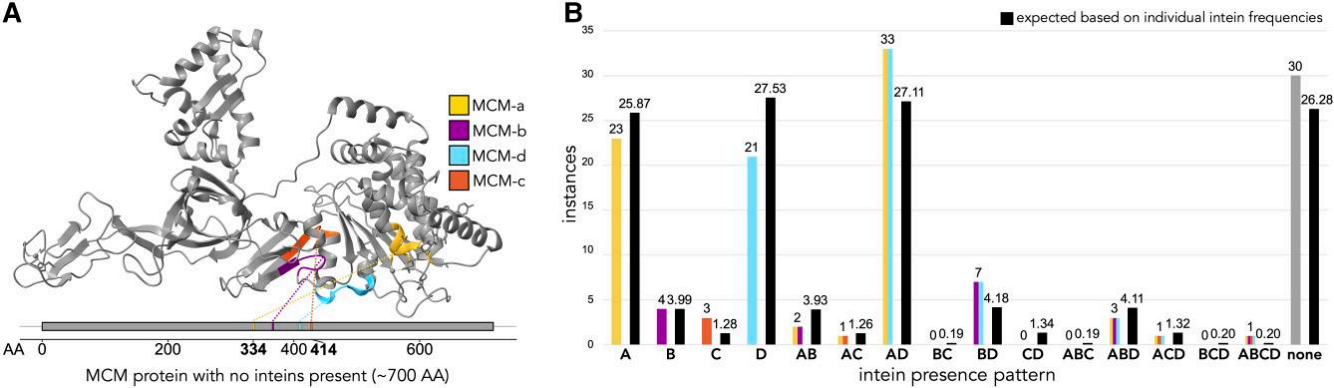
A) Structure of the MCM protein with the four intein insertion sites indicated. Note that the inteins are labeled in order of their discovery, and not by location in the linear sequence. Insertion site positions on the linear protein map are derived from *Haloferax volcanii* strain DS2 MCM (protein accession WP_004045334.1). The structure is from the homologous MCM hexamer of *Saccharolobus solfataricus* P2 (pdb: 4R7Ychain A) (18). B) Frequency of the four MCM inteins and their combinations in a set of 129 Haloferacales genomes. Black bars show the expected distribution of invasion patterns based on the total occurrences of each intein in the set overall, if the four inteins were independently and randomly distributed. *Hfx. mediterranei* WR646 harbors MCM-a and d, *Hfx. volcanii* SS0101 harbors only MCM-a, and *Hfx. volcanii* DS2 does not harbor any intein in the *mcm* gene.

The minichromosome maintenance (MCM) protein at the core of haloarchaeal replicative DNA helicase contains four active intein insertion sites (17) (Fig. 1A). Inteins at the two most frequently invaded insertion sites, MCM-a and MCM-d, will be referred to as double inteins. This double intein occurs more frequently in the Haloferacales than expected from the MCM-a and MCM-d frequencies (Fig. 1B). Here we explore the MCM double inteins in *Haloferax mediterranei* (*Hfx. mediterranei*) that contain LAGLIDADG/DOD family endonuclease domains (5, 19–21) and characterize their ability to cut their cognate DNA targets, as well as the homologous target sites from the distantly related species *Haloferax volcanii* (*Hfx. volcanii*). We show that each of the HEN domains on their own are highly active in cutting their respective target. Surprisingly, when both HENs are present, as is the wild-type situation, homing becomes highly inefficient, indicating difficulties in repairing two close double-strand breaks. This implies that having two active HENs will only be beneficial for horizontal propagation of the intein if there are single-intein alleles in the population, and a computational model supports this scenario. In agreement with the experimental findings and the theoretical model, an analysis of the distribution and phylogeny of the two *mcm* inteins revealed their frequent horizontal gene transfer but provided no evidence for their co-homing in Haloarchaea.

## Results

### Inteins in haloarchaeal *mcm*

The *mcm* gene in Haloarchaea has been invaded by inteins at four different insertion sites. InBase lists these inteins as CDC21-a, b, c, and d (22), though they may also be referred to with the MCM or MCM2 prefix. The MCM prefix reflects the revised naming of the host protein (16, 17, 23). The insertion sites of the MCM inteins are referred to as MCM-a, b, c, and d, respectively, and we will refer to the inteins using the same naming convention. For instance, the intein MCM-a invades at intein insertion site MCM-a. The MCM intein insertion sites are located close to one another both with respect to the host protein's structure and the linear sequence (Fig. 1A). All of the insertion sites fall within the conserved MCM helicase catalytic domain. In particular, the MCM-a insertion site is in the Adenosine triphosphate (ATP)-binding site following the conserved lysine in the Walker A motif, the same position where the Vacular Membrane ATPase (VMA) b intein is inserted in archaeal ATP synthases. The MCM-c insertion site also is part of the ATP-binding site, which is formed by two neighboring subunits in the MCM hexamer. The insertion sites for MCM-b and d line the channel through which the single-stranded DNA is pulled, when the two DNA strands are separated prior to replication (23). The most frequently occupied insertion sites in a set of 129 analyzed Haloferacales MCM orthologs are MCM-d (66 times out of 129) and MCM-a (64 times). Both inteins occurred together in 38 of the MCM orthologs, and in 33 of these instances, only the MCM-a and d inteins were present (Fig. 1B).

**Fig. 2.**
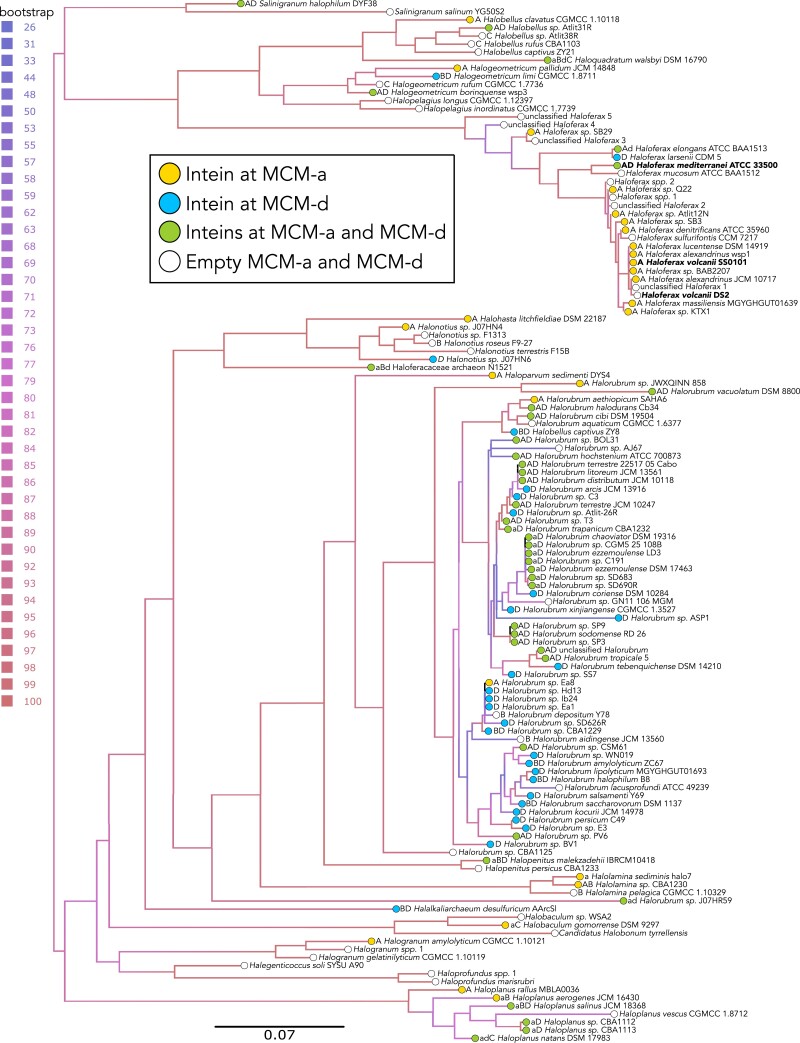
Phylogeny of MCM exteins from 129 Haloferacales strains. The presence and absence of the MCM-a and MCM-d inteins are indicated by colored circles in front of the species and strain designation and a prefix on each label. In each prefix, lowercase letters indicate mini-inteins and capitalized letters indicate full inteins. Of 64 MCM-a inteins, 19 had no detectable HEN sequence; of 66 total MCM-d inteins only five had no detectable HEN sequence. Phylogenies were calculated from the amino acid sequences using IQ-TREE (24) with ModelFinder. Support values were calculated from 1,000 ultrafast bootstrap samples. The model chosen using the Bayesian Information Criterion was LG + F + R6. The depicted phylogeny was rooted using minimal ancestor deviation (25). See Dataset S1 for the alignment of the MCM proteins in nexus formats and Dataset S2 for the phylogenetic tree in Newick format.

The phylogeny of MCM extein sequences within the Haloferacales (Fig. 2) is broadly compatible with the core haloarchaeal phylogeny based on concatenations of conserved proteins (26, 27). The original MCM set (n = 135) contained six viral *mcm* homologs, none of which contained inteins. In phylogenetic reconstruction, the viral homologs are grouped together on long branches. These divergent sequences lowered the support values for neighboring branches, and their grouping together might have been the result of long-branch attraction. Therefore, the viral homologs were removed from subsequent analysis of the final set (n = 129). Within the analyzed Haloferacales, 12 *Haloferax* strains harbor only MCM-a, one only MCM-d, two MCM-a and MCM-d, and 10 possess neither intein. Similarly, of the analyzed *Halorubrum* strains, three harbor only MCM-a, 23 only MCM-d, 26 both, and seven do not contain either intein. The distribution of intein presence and absence along the extein phylogeny indicates frequent loss and gain of the inteins. This is confirmed by comparing the extein phylogeny to the phylogenies calculated from the MCM-a and MCM-d sequences (Figs. S1 and S2). Several conflicts indicate between-species intein transfer. For example, MCM-a and MCM-d sequences from *Halorubrum* strains fall into two distinct clearly separated groups. MCM-d sequences from *Hfx. mediterranei* strain ATCC_33500 and *Hfx. larsenii* strain CDM-5 group together with an intein from *Halogeometricum*, whereas the MCM-d sequence from *Hfx. elongans* strain ATCC_BAA1513 forms a deeper branch.

### In vivo activity of MCM intein HEN domains

**Fig. 3.**
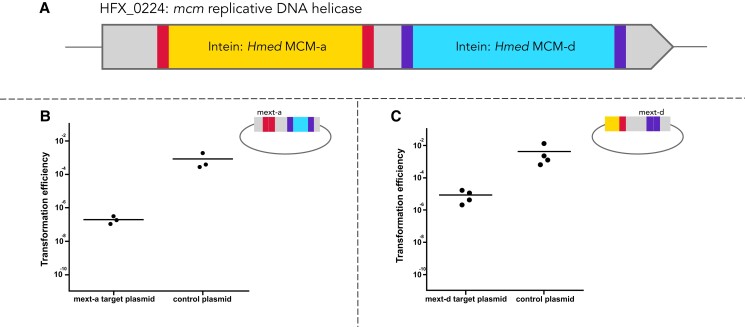
A) Schematic representation of the *mcm* gene in *Hfx. mediterranei* with the two inteins; *Hmed* MCM-a and *Hmed* MCM-d. The neighboring extein regions for both of the inteins are indicated in lighter (red) and darker (purple) colors respectively, mext-a, d. Transformation efficiency experiments of WR646 cells with a mext-a target plasmid (panel B) or a mext-d target plasmid (panel c) showed decreased efficiency in comparison to the control. The horizontal lines in B and C) correspond to the means.

We focused on the two inteins present in the *mcm* gene (HFX_0224) in *Hfx. mediterranei* strain WR646, derived from strain ATCC 33500, (*Hmed* MCM-a and d, see Fig. 2 and Figs. S1 and S2 for their phylogenetic distribution). To separately examine the activity of each HEN for both inteins, we constructed two individual plasmids which contained the insertion site portion of the extein for each intein; “mext-a” (*Hmed* MCM-a exteins, marked in gold, Fig. 3A) and “mext-d” (*Hmed* MCM-d exteins, marked in teal, Fig. 3A). These plasmids included additional flanking sequences derived from the gene. These extein sites also constitute the specific site that the HEN should recognize and cut. When *Hfx. mediterranei* cells that naturally contain the double MCM intein are transformed with either of these plasmids, one would expect “protection” from transformation (lower transformation efficiency) should the respective HEN domain be active, since in many cases cleavage of the target by the HEN will lead to plasmid degradation. Indeed, when such transformation experiments were carried out, we observed a decrease in the transformation efficiency of both mext-a and mext-d in comparison to the control plasmids, which do not contain a homing site (Figs. 3B and C). This suggests that both HEN domains can recognize and efficiently cleave their respective extein sequences. Both of these constructs also contained over 900 bp of homologous sequences from each side of their uninvaded extein sites that allow homologous recombination and consequently homing. Successfully transformed cells were screened for invasion of the corresponding intein to the target plasmid using PCR Using specific PCR primers flanking the multiple cloning site of the pTA230 plasmid (mext-a with *Hmed* MCM-a and mext-d with *Hmed* MCM-d). Most of the screened colonies showed elongation of the PCR product (14/14 colonies in mext-a, and 14/17 colonies in mext-d, Fig. S4) indicating that intein homing occurred in both cases. Plasmids were extracted from *Hfx. mediterranei* cells and transformed into *E. coli* and then extracted again. Plasmid DNA was used for Sanger sequencing using internal primers IS542 and IS668 that match the *Hfx. mediterranei* mcm gene in the downstream and upstream of the inteins integration sites. The plasmids were shown to contain the respective intein sequences (see Fig. S9).

### Both HEN domains in MCM can recognize and cleave target sequences from distant Haloferax species

**Fig. 4.**
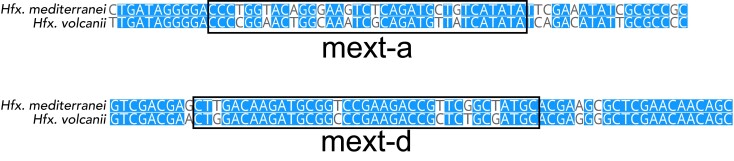
Sequence alignment of the mext-a and mext-d sites in *Hfx. mediterranei* and *Hfx. volcanii*. A high similarity between *Hfx. volcanii* and *mediterranei* in the extein sequence of the *mcm* gene can be observed.

**Fig. 5.**
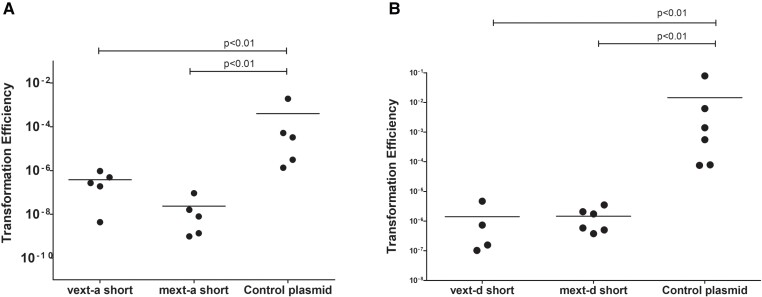
Transformation efficiency experiments of WR646 cells with A) a vext-a short and a mext-a short target plasmid or B) a vext-d and a mext-d target plasmid, both having Hfx. volcanii DS2 extein sequences. Significant decrease in transformation efficiency in comparison to the control (target-less) plasmid (pTA230) was observed, indicating endonuclease activity. The horizontal lines in both A and B corresponds to the means. In addition, different samples were compared by a two-sided Mann–Whitney U test, *P* < 0.01.

It has been suggested that one of the selective forces maintaining the activity of HEN domains is the ability to invade other species and populations through horizontal gene transfer. If this is indeed a possibility, one would expect HEN domains to recognize homologous allele sequences from remote species of the same genus. One such strain is *Hfx. volcanii* DS2, which has an uninvaded *mcm* gene that represents a natural target for the *Hfx. mediterranei* MCM HENs. This nucleotide sequence has several substitutions (most of them synonymous) compared to the *Hfx. mediterranei* allele (Fig. 4). We note that a different strain from the same species, *Hfx. volcanii* SS0101, harbors the MCM-a intein. We constructed target plasmids with short *Hfx. volcanii* MCM extein (vext-a short and vext-d short) sequences and tested whether they will be recognized by the *Hfx. mediterranei* MCM HENs using the plasmid protection assay, as above. As a control, we also constructed short *Hfx. mediterranei* target plasmids (mext-a short and mext-d short), with only minimal target sites but without homologous flanking regions that allow homologous recombination and homing, which in *Haloferax* should lead to repair via microhomology-mediated end joining (28, 29). As was observed for its own cognate site, the transformation efficiency was lower when the target plasmid contained the vext-a that is homologous to the mext-a sequence (Fig. 5A), as well as when it contained vext-d that is homologous to mext-d (Fig. 5B). These results indicate that the HEN domains of the *Hfx. mediterranei* MCM are highly active and can recognize and cleave homologous sequences from other species of *Haloferax*. This is in agreement with previous work showing that LAGLIDADG HENs can tolerate substitutions in their homing site and cleave sites from related microbial species (28, 30, 31). We scanned 20 transformed colonies from each experiment (using one of the four plasmids), using PCR with primers flanking the extein site on the plasmid, looking for elongated fragments in a DNA gel that could indicate intein invasion. However, no intein invasion could be detected. This could be due to the lack of an adequately long fragment for homologous recombination to occur, required for intein invasion, since the extein sequences provided were very short (34–35 bp), which may be too short for efficient homologous DNA recognition and recombination.

We then sequenced the plasmids from five colonies of each transformation experiment (mext-a short, mext-d short, vext-a short, vext-d short) using Sanger sequencing and observed “escaper” mutations in the extein sequences on the plasmids that had been successfully transformed in both mext-a and vext-a plasmid. In the mext-a plasmids, we could detect either a deletion of the entire extein site (2/5) or point mutations (3/5). In the “vext-a” homolog, we observed point mutations in two out of five cases, whereas 3/5 had intact extein sequence, indicating that there may be decreased HEN activity against the homologous sequence that may enable the retention of the wild-type target (Fig. S5). In 5/5 of the mext-d and 5/5 of the vext-d derived plasmids we were able to observe shortening of the plasmids in comparison to the original insert-containing plasmid using agarose gel DNA electrophoresis (Fig. S6). This finding indicates DNA damage and loss of DNA due to microhomology-mediated end joining: a mechanism of DNA repair that operates in *Haloferax* species when there is a lack of sufficient homologous sequences to allow homologous recombination. Thus, the HEN activity against the original and homologous extein sequence apparently led to DNA damage and repair.

### HEN-mediated homing of the MCM double inteins into an intein-less allele is highly inefficient

**Fig. 6.**
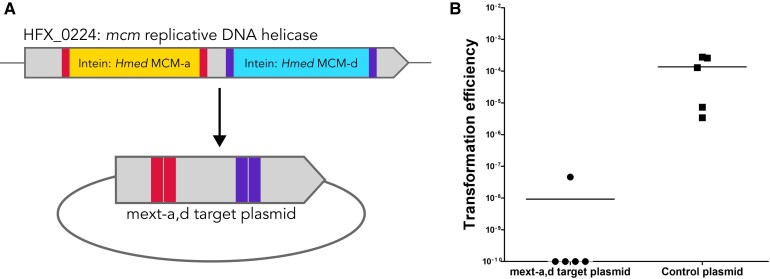
A) Schematic representation of the *mcm* gene in *H. mediterranei* with the two inteins, *Hmed* mcm-a *Hmed* mcm-d. The insertion site regions for both inteins are indicated on the extein in lighter (red) and darker (purple) color, respectively. The arrow points to the shuttle vector built to have most of the *mcm* gene without the inteins. B) Transformation efficiency of *Hfx. mediterranei* WR646 cells, containing MCM inteins, using the shuttle vector built to have most of the *mcm* gene without the inteins, mext-a, d target plasmid, shows decreased efficiency in comparison to the control (pTA230). The horizontal lines correspond to the means.

**Fig. 7.**
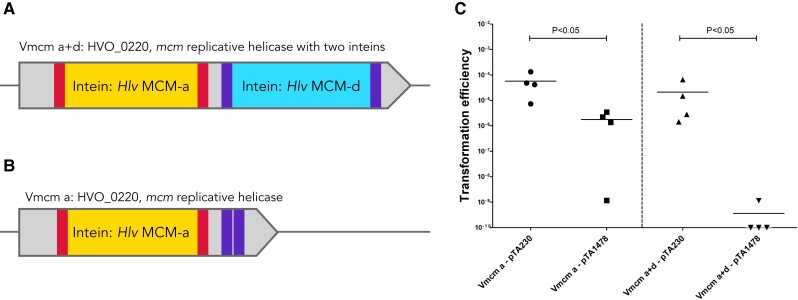
A) Schematic representation of the *mcm* gene in the *H. volcanii* strain with the two inteins, Vmcm_a+d. The extein regions for both inteins are indicated in lighter (red) and darker (purple) color, respectively. B) Schematic representation of the *mcm* gene in the *H. volcanii* strain with one intein, Vmcm_a. C) Transformation efficiency of *Hfx. volcanii* Vmcm_a+d and Vmcm_a, using shuttle vector pTA1478 with the mcm gene without inteins, and a control plasmid that has no targets (pTA230). The horizontal lines correspond to the means. In addition, different samples were compared by a one-sided Mann–Whitney U test. The *P*-value is provided in each case.

Since either HEN could efficiently mediate homing into an intein-less allele when the other site contained an intein, we tested the ability of the wild type double MCM intein to invade an empty *Hfx. mediterranei* site, analogous to the natural *mcm* allele of *H. volcanii.* The empty target was 1931bp long and therefore had sufficiently long homologous sequences to enable homologous recombination (Fig. 6A). However, when this target plasmid was transformed into *Hfx. mediterranei*, no colonies could be obtained in 4/5 biological replicates (Fig. 6B). In the single replicate that did result in transformed *Hfx. mediterranei*, only two colonies were obtained. Sequencing of the plasmids from these colonies showed that the two inteins successfully invaded both sites. We wanted to further examine the efficiency of intein invasion of two inteins with active HENs into adjacent sites in comparison to single site intein invasion. However, attempts to cure either intein from *Hfx. mediterranei* were unsuccessful. Therefore, we constructed two *Hfx. volcanii* strains with inteins in the *mcm* helicase gene (HVO_0220); “Vmcm_a+d”, with the two inteins (originally from *Hfx. mediterranei)*, and “Vmcm_a”, with only one intein (Fig. 7A and B).

Our analysis of MCM-d insertion sites (Figs. S3 and S7 and below) suggested that *mcm* genes that encode a proline downstream of the intein insertion site are resistant to intein invasion. Indeed, as expected, attempts to generate the intein-containing *mcm* allele with the original proline extein repeatedly resulted in no successful allele exchange, indicating that an *mcm* allele with the intein followed by proline in the extein was not viable (see methods). Therefore, we changed the extein sequence to a serine instead of the original *H. volcanii* proline to allow protein splicing to occur via nucleophile displacement, which requires either serine, cysteine, or threonine (29).

We transformed the Vmcm_a+d and Vmcm_a *H. volcanii* strains with pTA1478: a plasmid containing the empty *Hfx. volcanii mcm* allele with approximately 2Kb flanking sequence from each side of the gene, enabling repair by homologous recombination. As expected, transformation was more efficient using the control plasmid, without homing sites, than using the pTA1478 plasmid in both strains, Vmcm_a and Vmcm_a+d (Fig. 7C). Transformation using the Vmcm_a strain was lower by about one order of magnitude than the control, whereas the Vmcm_a+d transformation efficiency was five orders of magnitude lower than the control, with 3/4 biological replicates resulting in three colonies. All showed both a and d intein invasion (Fig. S8). Transformed colonies from the Vmcm_a experiment were screened for an intein invasion to the pTA1478 plasmid and showed a partially invaded genotype; all colonies contained cells with uninvaded plasmids as well as invaded plasmid, as detectable by PCR. Partial invasion of plasmids indicates that the plasmids most likely have been able to replicate in the host cell before being invaded. It is likely that with time all colonies will show full invasion. These results confirm the observations in *Hfx. mediterranei* (Fig. 3) that unlike single intein homing, double-intein homing into a completely intein-less target allele is highly inefficient, probably due to an inability to repair two double-strand breaks that are close to one another via homologous recombination.

### Phylogenetic analyses do not support co-homing of MCM-a and MCM-d

**Fig. 8.**
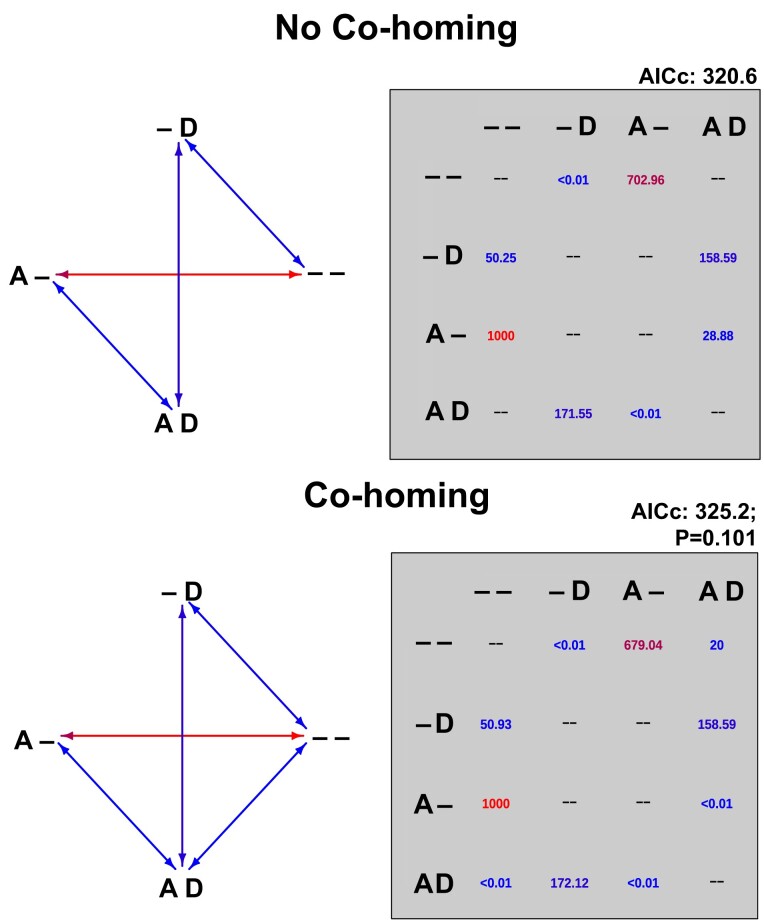
Suitability of intein homing models with and without co-homing. The two models evaluated are described in the arrow diagrams on the left. The arrows indicate state transitions possible in each model, while the color of the arrow corresponds to the calculated magnitude of the instantaneous transition rate. The possible character states for each taxon are coded as follows: – –: no intein in A site nor in D site; – D: intein in D site, but not in A site; A – intein in A site, but not D site; A D: both A and D sites have been invaded by intein. The matrices for both models show the calculated rates for each state transition in the model rate matrix. Cells that read—are considered invalid transitions for each model. The rates in the matrices are expressed as the “number” of transitions per unit of branch length. In this case, the branch length corresponds to the MCM extein tree (Fig. 2). The matrices should be read from **row** to **column**, the row being the starting state and the column being the end state (i.e. second row first cell is indicating the rate of transitioning from (− D) to (− −) or loss of D). Both evaluated models consider the possibility of sequential homing. For example, the cell at the third row and fourth column has a value of <0.01, this reports the number of transitions when the A site is occupied and the D site is empty (A–) to an allele which contains both inteins (AD).

Nature has provided us with a large-scale experiment of MCM-a and MCM-d homing in Haloferacales. To test if co-homing of two neighboring inteins occurred frequently in evolutionary history, we used the rooted extein phylogeny (Fig. 2) and the distribution of presence/absence data for the MCM-a and MCM-d inteins at the leaves of the phylogeny to fit two maximum likelihood models (32) for intein loss and gain. One model allowed only for the gain or loss of a single intein at a time, while the other model allowed for single or double homing and loss (Fig. 8). According to the corrected Akaike Information Criterion (AICc), the model without double homing is preferred: no co-homing AICc = 320.60, with co-homing AICc = 325.20, i.e. the more complicated model (with co-homing) has a relative likelihood of only 0.101 (P_model I_ = exp((AICc_min_ − AICc_i_)/2). While the co-homing model is not significantly rejected, the data do not support the more complex co-homing model. Co-homing, if it occurred at all, was an infrequent event in the evolutionary history of the *mcm* gene in the Haloferacales. In the few instances where we experimentally observed the two inteins invading a previously uninvaded target sequence, it is possible that the two inteins did not co-home, but rather invaded their respective target sites sequentially.

### Penetrance of the MCM inteins in Haloferacales and conservation of intein insertion sites

It is difficult to reconcile the observation that the primary means of generating an allele with only the MCM-d intein is through a loss of the MCM-a intein in an organism that had acquired both inteins (Fig. 8, co-homing model). However, analyses of the insertion sites of both intein alleles shed light on the intein transmission dynamics in Haloferacales (Figs S3 and S7). The insertion site for MCM-a is conserved throughout all sampled Haloferacales (i.e. in both invaded and uninvaded populations). Thus, the HEN in the MCM-a seems able to recognize all of its insertion sites in Haloferacales, and its calculated invasion rate reflects this (Fig. 8). Conversely, a clear dichotomy exists between the invaded and uninvaded insertion sites of the MCM-d intein: those invaded by an intein always encode a serine (S) immediately downstream of the insertion site, while uninvaded sequences primarily encode a proline (P). Given our results when artificially generating this intein and the known mechanism of protein splicing via nucleophile displacement, it is reasonable to assume that proline-containing sequences mostly represent previously uninvaded alleles. In addition, the residues flanking the MCM-d intein insertion site show more variation than MCM-a, particularly the first residue on the N-terminal side of the insertion site and the first two residues on the C-terminal side of the insertion site. Barring the previously mentioned serine (S) immediately downstream in all MCM-d intein-containing copies, this region is variable across all *mcm* copies in the set regardless of whether the MCM-d intein is present or absent (Fig. S7).

## Discussion

### Benefits and costs due to inteins

Inteins are usually located in conserved proteins, especially those involved in nucleotide binding, DNA metabolism, replication, and repair (6, 7). Specifically, they occupy conserved sites within these proteins (7), possibly because this facilitates homing into homologous proteins from other species, or because the intein can only be removed from the gene by a precise deletion (9). However, reliance on a small set of genes has limited the potential intein targets during evolution and has led to the situation where different inteins target different sites within the same gene, each using its own highly sequence specific HEN domain. In euryarchaea, where inteins abound, this constraint has resulted in a frequent occurrence of multiple inteins within the same gene, which we have explored here.

It has been suggested that inteins in proteins involved in replication, transcription, and translation might have acquired a regulatory function (6, 13, 33, 34). If an intein has acquired a function that provides a selective advantage to the host organism, the intein should become fixed in the population. While inteins may be beneficial under specific stresses, the observation that inteins such as MCM-a and d are frequently lost and gained in lineages (Fig. 2) speaks against these inteins having acquired a function that provides a sweeping selective advantage to the host under most environmental conditions, which would have made intein-less genotypes become extinct.

Naor et al. (35) showed that in two isogenic *Haloferax* strains where the only difference is the presence of the *Hfx* polB-c intein, a 7% fitness cost was associated with harboring the intein. Although different inteins alleles will differ in their effect on the host organism's fitness, double and triple inteins have a higher potential to represent a fitness cost than single ones. A selfish genetic element persisting in a lineage for a longer time will have more opportunities to acquire an additional function beneficial to the host. In the case of split inteins (36), the splicing domains have become an integral part of assembling the DNA replication machinery. This dependence on a complex assembly process of the DNA polymerase provides an illustration of constructive neutral evolution (37). The integration into conserved sites might guard the intein against deletion; however, loss of inteins does occur (Fig. 2) and according to the estimated model parameters (Fig. 8) occurs frequently.

### Intein benefits to neighboring DNA sequences

During homing, not only the intein-encoding DNA but also stretches of neighboring DNA may be incorporated into the recipient genome. This is illustrated by the observation that the MCM-d inteins form two clans in phylogenetic reconstruction based on the intein sequences (Fig. S3A), whose N-extein ends with aspartate or arginine, respectively. In contrast to the clear separation of the intein sequences, in the extein phylogeny, the two clans are not clearly separated, suggesting the inteins when moving into a new genome sometimes take at least the last codon of the N-extein with them (*c.f. Halorubrum vacuolatum* DSM 8800 and *Halopenitus malekzadheii* IBRCM10418 in Fig. S3B). Selfish genetic elements, even if they might not provide increased fitness to the host organism, can provide a selective advantage to neighboring DNA. Furthermore, intein splicing requires specific amino acids at the splice site, which sometimes forces part of the extein to invade as well, as in the case of conversion of proline to serine by the MCM-d intein (see below).

### Interactions between neighboring inteins

John Avise (38) discussed metaphors for interactions between genetic elements normally associated with species in natural biological communities. The interactions between an intein and a host gene then can be described as parasitism. Targeting the same conserved regions was suggested as a mechanism through which self-splicing group I introns and homing endonucleases evolve into a single selfish genetic element (39). Before the work reported here, a reasonable expectation was that neighboring inteins might be in a mutually beneficial symbiotic relationship, similar to the co-homing model in several selfish introns (40, 41). However, we find exactly the opposite. Both inteins, MCM-a and d, were shown to have functioning HENs that recognized target sequence from the same genome (i.e. the DNA sequence surrounding the insertion sites in *Hfx*. *mediterranei*) and the target sites from the genome of the distantly related *Hfx*. *volcanii* DS2 (Fig. 4); however, the HENs initiated homing only when a single empty target site was available (Figs. 5–7). Our experiments revealed that the existence of two functioning HENs that target nearby sites prevented efficient homing. If this observation can be generalized, neighboring inteins with functioning HEN form a partial dead end: MCM-a plus MCM-d plus inteins can invade MCM-a plus MCM-d minus or MCM-a minus MCM-d plus alleles, but much less frequently or not at all, recipients that are MCM-a minus MCM-d minus. They can only occur together in one gene if there are many single-invaded alleles in the population and in both potential invasion sites.

Our phylogenetic analyses arrived at the same conclusion. We did not find evidence for frequent co-homing of the neighboring MCM-a and MCM-d inteins. Both inteins do occur and co-occur frequently in Haloferacales. Their presence/absence mapped onto the extein phylogeny (Fig. 2) and the comparison between extein and intein phylogenies (Figs. S1 and S2) reveals the frequent transfer of these inteins between strains and species. However, fitting the intein distributions onto the extein tree using different maximum likelihood models did not reveal a significant contribution of co-homing (Fig. 8).

The extent to which our observations about the in vivo activity of the *mcm* intein in *Haloferax* can be generalized to most other genes and species will require further research. The presence of a HEN domain in a similar fraction of double inteins as that observed for single-intein genes in the same taxonomic groups (Table 1), suggests that in general HENs in double inteins are as likely (or rather unlikely) as single intein HENs to lose their mobility. In contrast, the magnitude of homing difficulties of double inteins into vacant alleles that we report here for *Hfx. mediterranei*, can be host-specific, since it will depend on how efficiently both homologous recombination (HR) and strand resection operate at double-strand breaks in the species in question. It will be intriguing to experimentally test double-intein homing in the Thermococcales, where double inteins are very common and in which genetic systems exist (42, 43).

Unlike the situation in *Hfx. mediterranei*, in other cases of double inteins, such as the *mcm* gene of the hyperthermophilic archaeon *Thermococcus kodakarensis* (43) or its bacterial analog, the *dnaB* gene of *Mycobacterium smegmatis* (44), one intein is a mini-intein devoid of a HEN domain that thus must rely on co-homing, if it homes at all. It may be that in the *Hfx. mediterranei mcm* gene both HEN domains are required for intein splicing, as observed previously for the *Hfx. volcanii polB* intein (45), preventing either of them from degenerating to mini-inteins, and explaining why HEN domains are found in a similar fraction of single and double inteins in haloarchaea (Table 1). Nonetheless, the *Hfx. elongans* mcm intein D appears to have lost its HEN domain, so clearly, loss of this HEN domain may be difficult but not impossible. Further research is needed to explore how frequently an intein without an HEN domain can home with the help of a HEN domain in a neighboring intein.

### *mcm* alleles resistant to invasion

An initially puzzling observation was the near-negligible rate of MCM-d invasion (Fig. 8). In contrast to the MCM-a insertion site, which is highly conserved (Fig. S7) the insertion site targeted by MCM-d contains several variable positions. Most empty target sites encode a proline following the insertion site, whereas occupied sites encode a serine in this position (Figs. S3B and S7). The MCM-d intein sequences observed in nature are inserted between either R/S or D/S. The resistance to invasion is not due to an inability of HEN to recognize the target site (Fig. 5B), but may rather reflect the proline downstream of the extein, which prevents the successful splicing reaction (31). Given the essentiality of MCM, this proline needs to be co-converted into a serine during intein invasion. Mapping the amino acids flanking the insertion site onto the extein phylogeny (Fig. S3B) reveals a few invasions via co-conversion of the flanking proline; however, the rate for these co-conversion events is low compared to the MCM-a invasion rate (Fig. 8).

## Conclusion

Taken together, the modeling of natural intein distributions and our experimental results suggest a situation in which the double intein can almost exclusively invade singly invaded sites, but not empty alleles. While this goes against the expectation of co-homing, it provides a better explanation for the distribution of *mcm* allele sequences observed in extant Haloferacales. It explains at least partly why there are a relatively high amount of single-intein alleles in this order—if double invasions were more efficient, one might expect a situation where all alleles in the population are either doubly invaded or noninvaded, and this is not the case. The double intein thus represents “the end of the road” for the two inteins involved, and should it encounter a noninvaded population of closely related Haloferax strains, it is expected to go extinct, only to remerge when two strains that have MCM inteins at different (single) sites come into contact.

## Materials and methods

### Strains and plasmids

Strains described in Table S1, plasmids in Table S2, and oligonucleotides in Table S3.

### Culture conditions

*Hfx. volcanii* and *Hfx. mediterranei* cells were routinely grown as described in (46).

### *Haloferax* strain construction

*Hfx*. *volcanii* strains that contain *Hfx. mediterranei* intein insertions (intein a and inteins a+d) in their *mcm* genes were generated using pop-in pop-pout allele exchange, according to the protocol described in (47, 48). The Vmcm_a+d strain was constructed in two steps; intein a was inserted first and then intein d was successfully added only after the extein region was also altered- replacing serine with proline in position 401 of the protein, which the position of the extein that flanks the C-terminus of the intein. Notably, attempts to generate this intein while maintaining the original proline were unsuccessful, despite successful invasion at the pop-in stage, since no pop-out colonies that had the intein could be obtained.

### Transformation

Transformation of *Hfx. volcanii* and *Hfx. mediterranei* was carried out using the polyethylene glycol (PEG) 600 method as described in (47, 49). 1.5 ml of liquid culture was grown in *Haloferax volcanii* yeast peptone casamino (Hv-YPC) medium to OD_600nm_ of 1.5, and then centrifuged at 3,600*×g* for 5 minutes. The supernatant was discarded, and the cells were resuspended in 200 µl spheroplasting solution (1 M NaCl, 27 mM KCl, 50 mM Tris–HCl PH 8.5, 15% sucrose) and incubated at room temperature for 5 minutes. 20 μl of 0.5 M ethylenediaminetetraacetic acid (EDTA) were added and cells were incubated at room temperature for 10 minutes. 10 µl of purified plasmid DNA containing the selective marker uracil (approximately 250 ng/µl) were mixed with 15 µl spheroplasting solution and 5 µl of 0.5 M EDTA, and added to the cells, followed by incubation of 5 minutes at room temperature. Subsequently, 250 µl of PEG solution (60% PEG 600 [Sigma-Aldrich] in spheroplasting solution) was added, and cells were incubated for 30 more minutes at room temperature. Following the incubation, 1 ml of regeneration solution (3.4 M NaCl, 175 mM MgSO_4_, 34 mM KCl, 5 mM CaCl_2_, 50 mM Tris–HCl pH 7.5, 15% sucrose) was added and cells were centrifuged at 6,000 rpm for 7 minutes. The supernatant was discarded, and cells were resuspended in an Hv-YPC medium supplemented with 15% sucrose and left to incubate without shaking overnight at 37°C. The cultures were then transferred to a 37°C shaker and left for an incubation of 3 more hours, then washed and plated on selective media. Colonies that successfully absorbed the plasmid could grow on selective media lacking uracil (Hv-Ca).

### Quantifying transformation efficiencies

Assessing transformation efficiency of *Hfx. volcanii* and *Hfx. mediterranei* strains (Table S1) transformed with target plasmids containing extein sequences and a control plasmid pTA230 (Table S2) was calculated as the number of successfully transformed colony forming units (CFUs) grown on selective plates (Hv-Ca) divided by the number of CFU grown on nonselective plates (Hv-YPC).

### Scanning for intein invasion

The different extein target plasmids (Table S2) were transformed into *Hfx. mediterranei* and *Hfx. volcanii* cells. Colonies positive for the selective marker (uracil) were scanned for intein invasion by PCR (Phusion, Thermo Scientific, USA), using primers designed to anneal to the multiple cloning site of the target plasmid, surrounding the DNA insert. invasion sites of the target plasmids (Table S3). Observation of elongated PCR products indicated intein invasion. For short target plasmids the PCR products were sent for Sanger sequencing with primers IS498 and IS499. In order to sequence the invading long inteins in the mext-a and mext-d plasmids we first extracted the plasmids from the archaeal cells and then transformed them to *E. coli* and plated the bacteria on Luria-Bertani (LB) medium with ampicillin (the selectable marker for these plasmids). Then, plasmids were extracted from *E. coli* and Sanger-sequenced using IS542 and IS668.

### Phylogenetic inference

Extein and intein sequences were curated in SeaView and aligned with MUSCLE. Due to variability with respect to intein presence or absence at the four insertion sites, a guided approach was taken: sequence sets sharing intein presence patterns were individually aligned using MUSCLE and gaps matching the full intein size at each uninvaded site were added. For example, the set containing all sequences with inteins at MCM-a and MCM-d were assigned intein-sized gaps at sites MCM-b and MCM-c. All sets were then combined and realigned together with MUSCLE once more to unify the alignment. This approach allowed distinct intein and extein bounds to be established in SeaView. Phylogenies were then inferred using the best model as determined by the Bayesian Information Criterion (BIC) in IQTree. Node support was estimated by using 100 nonparametric bootstrap samples for the intein trees, and 1,000 samples of ultrafast bootstrap for the extein tree. The minimal ancestor deviation algorithm (25) was used to root every tree to ensure accuracy and universal consistency for topological comparisons.

### Intein insertion site analyses

Twenty amino acid residues upstream/downstream of each intein insertion site, invaded, and uninvaded, were extracted for comparison. Alignment subsets were created based on each intein allele (MCM-a/d) and whether or not that extein had been invaded (i.e. MCM-a invaded/MCM-a uninvaded). Weblogo (50) was used to generate graphic representations of the composition of residues at each site (Fig. S7). In addition, the amino acids flanking the insertion site were mapped onto the extein phylogeny (Fig. S3B).

### Detection of multiple intein-containing genes

Sequences from the genomes containing inteins were gathered using 25 Position Specific Scoring Matrices (PSSMs) of the known inteins found in Haloarchaea (completely sequenced haloarchaeal genomes available at NCBI on March 06, 2020) and the number of intein hits per sequence were counted. These sequences were then searched again with PSSMs made from the alignment of the 4 LAGLIDADG HEN blocks commonly found in inteins. When HEN blocks were detected, the intein was categorized as a full intein, when absent they are counted as mini-inteins.

### Co-homing enabled vs prohibited models

The two models were characterized by their transition rate matrices (Fig. 8) and were identical except the model with co-homing allowed for the simultaneous the gain and loss of two adjacent inteins. The MCM extein phylogeny from Fig. 2 was used as the reference phylogeny in the corHMM function (32). The character state information of each taxon corresponds to the labeling in Fig. 2. The AICc and transition rates (Fig. 8) were calculated in corHMM, using the above as input, and with the default parameters (excluding the upper bound of the likelihood search, which was increased to 100,000).

## Supplementary Material

pgad354_Supplementary_Data

## Data Availability

All data is included in the manuscript and supporting information.
