# Supplementary material for: Neighboring inteins interfere with one another's homing capacity
Source: PNAS Nexus. 2023 Oct 27;2(11):pgad354. doi: 10.1093/pnasnexus/pgad354 (PMC10643990; doi:10.1093/pnasnexus/pgad354)
Supplement: pgad354_Supplementary_Data [file pgad354_supplementary_data.zip › PNASNEXUS-PNASNEXUS-2023-00472RR-s01.pdf]

PNAS nexus

Supplementary Information for

Neighboring inteins interfere with one another's homing capacity.

Israela Turgeman-Grott[1], Danielle R. Arsenault[2], Dekel Yahav[1], Yutian Feng[2], Guy Miezner[1], Doron Naki[1], Omri Peri[1], R. Thane Papke[2], Johann Peter Gogarten[2,3,*], and Uri Gophna[1,*].

1: The Shmunis School of Biomedicine and Cancer Research, Faculty of Life Sciences, Tel Aviv University, P.O. Box 39040, 6997801, Tel Aviv, Israel

2: Department of Molecular and Cell Biology, University of Connecticut, Storrs, CT 06268-3125, USA

3: Institute for Systems Genomics, University of Connecticut, Storrs, CT 06268-3125, USA

*: Corresponding authors:

Johann Peter Gogarten, Department of Molecular and Cell Biology, University of Connecticut, 91 North Eagleville Rd, Storrs, CT 06268-3125, USA; Phone 1 860 465 6267;
E-mail: gogarten@uconn.edu.

Uri Gophna, The Shmunis School of Biomedicine and Cancer Research, Faculty of Life Sciences, Tel Aviv University, 6997801, Tel Aviv, Israel;
E-mail: urigo@tauex.tau.ac.il

This PDF file includes:

Figures S1 to S9
Tables S1 to S3 (not allowed for Brief Reports)
Legend for Datasets S1-S2
SI References

Other supplementary materials for this manuscript include the following:

Dataset S1
Dataset S2

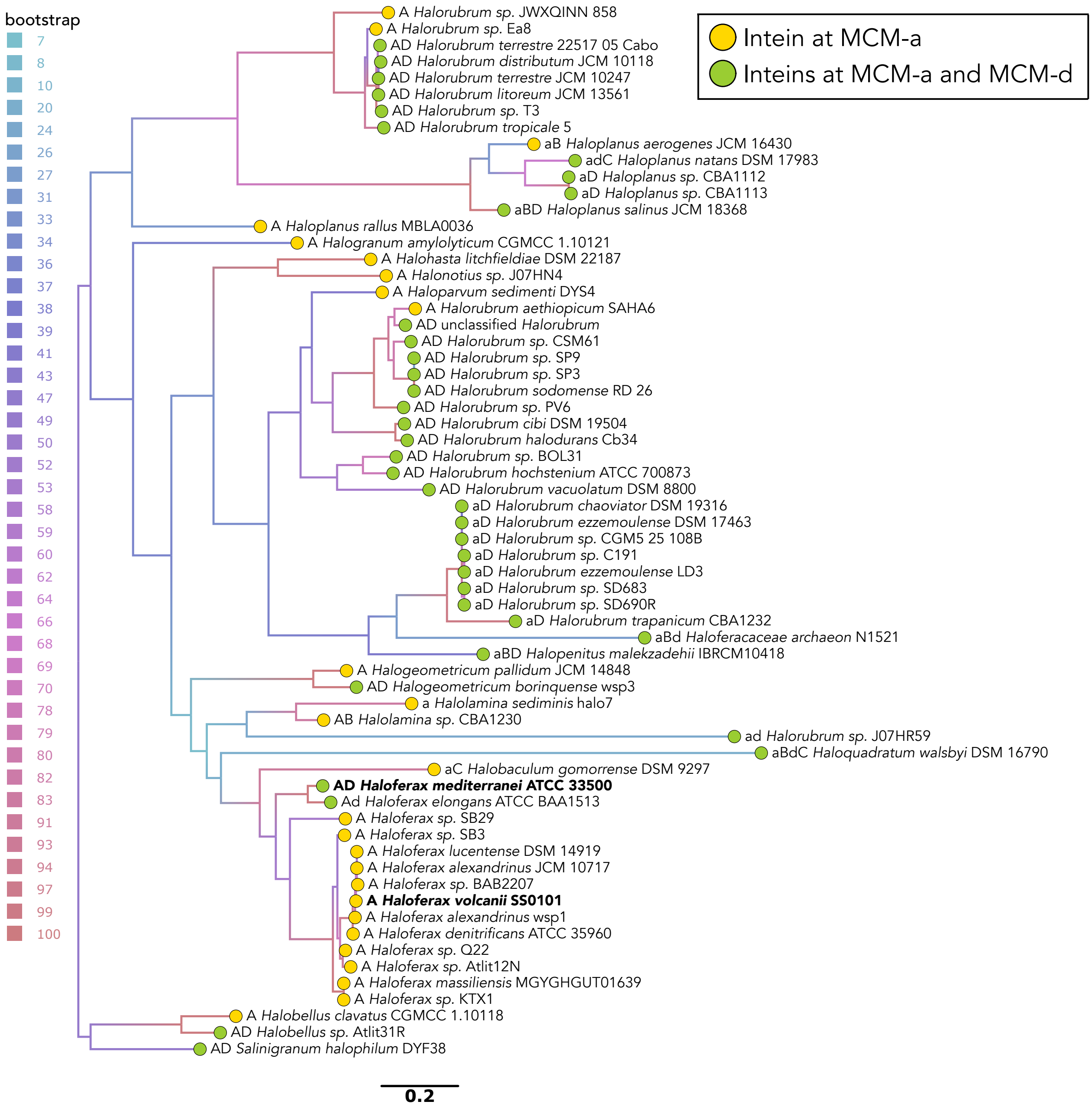

Fig. S1: Phylogeny of MCM-a inteins. Phylogenies were calculated from the amino acid sequences using IQ-TREE (1) with ModelFinder. Support values were calculated from 100 traditional bootstrap samples. The chosen model was LG+F+I+G4. The phylogeny should be considered unrooted. See Supplementary Dataset S2 for the tree file in computer readable Newick format, and Supplementary Dataset S1 for the partitioned alignment of the MCM proteins in nexus format.

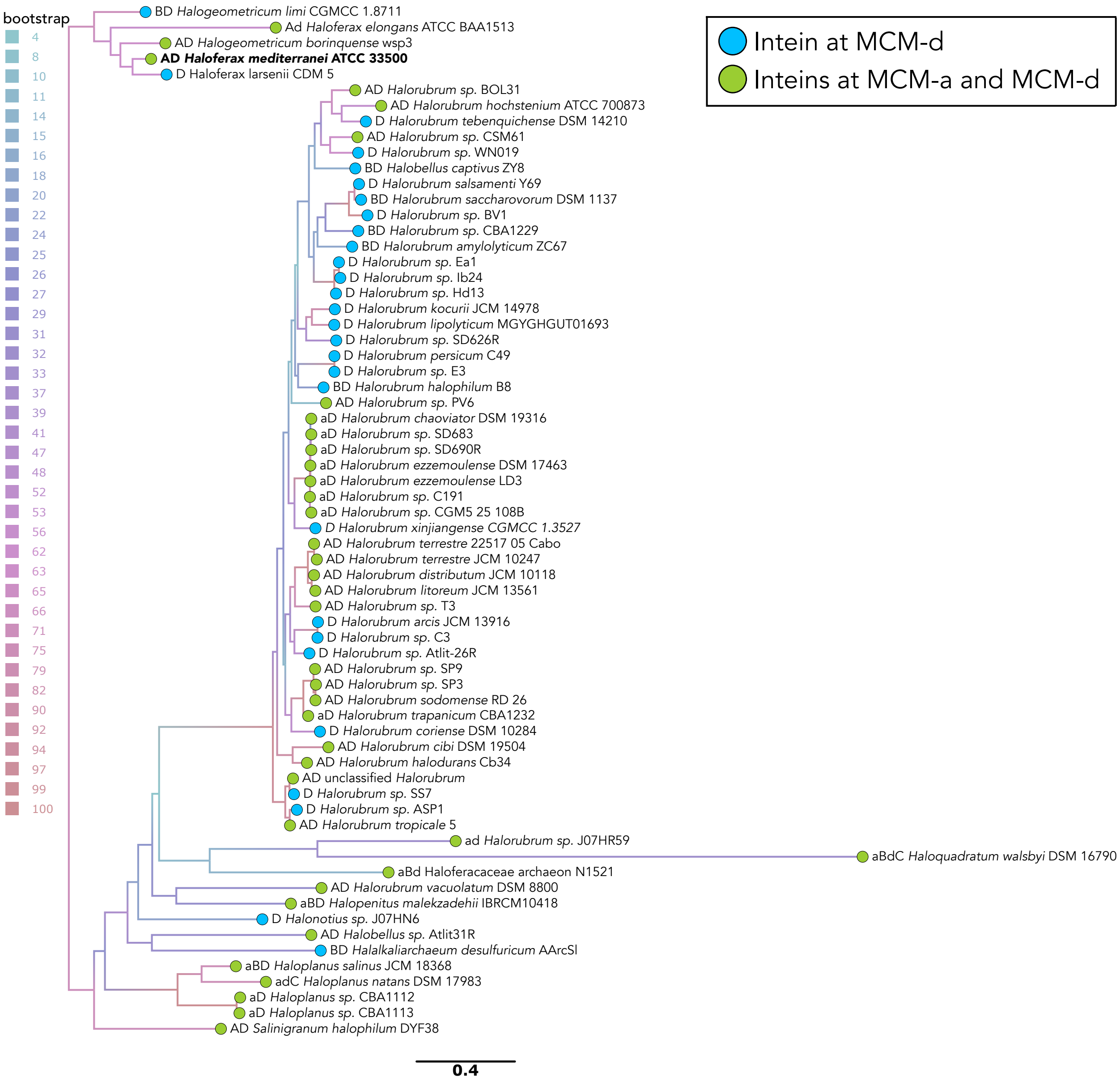

Fig. S2: Phylogeny of MCM-d inteins. Phylogenies were calculated from the amino acid sequences using (1) with ModelFinder. Support values were calculated from 100 traditional bootstrap samples. The chosen model was LG+F+I+G4. The phylogeny should be considered unrooted. See Supplementary Dataset S2 for the tree file in computer readable Newick format, and Supplementary Dataset S1 for the partitioned alignment of the MCM proteins in nexus format.

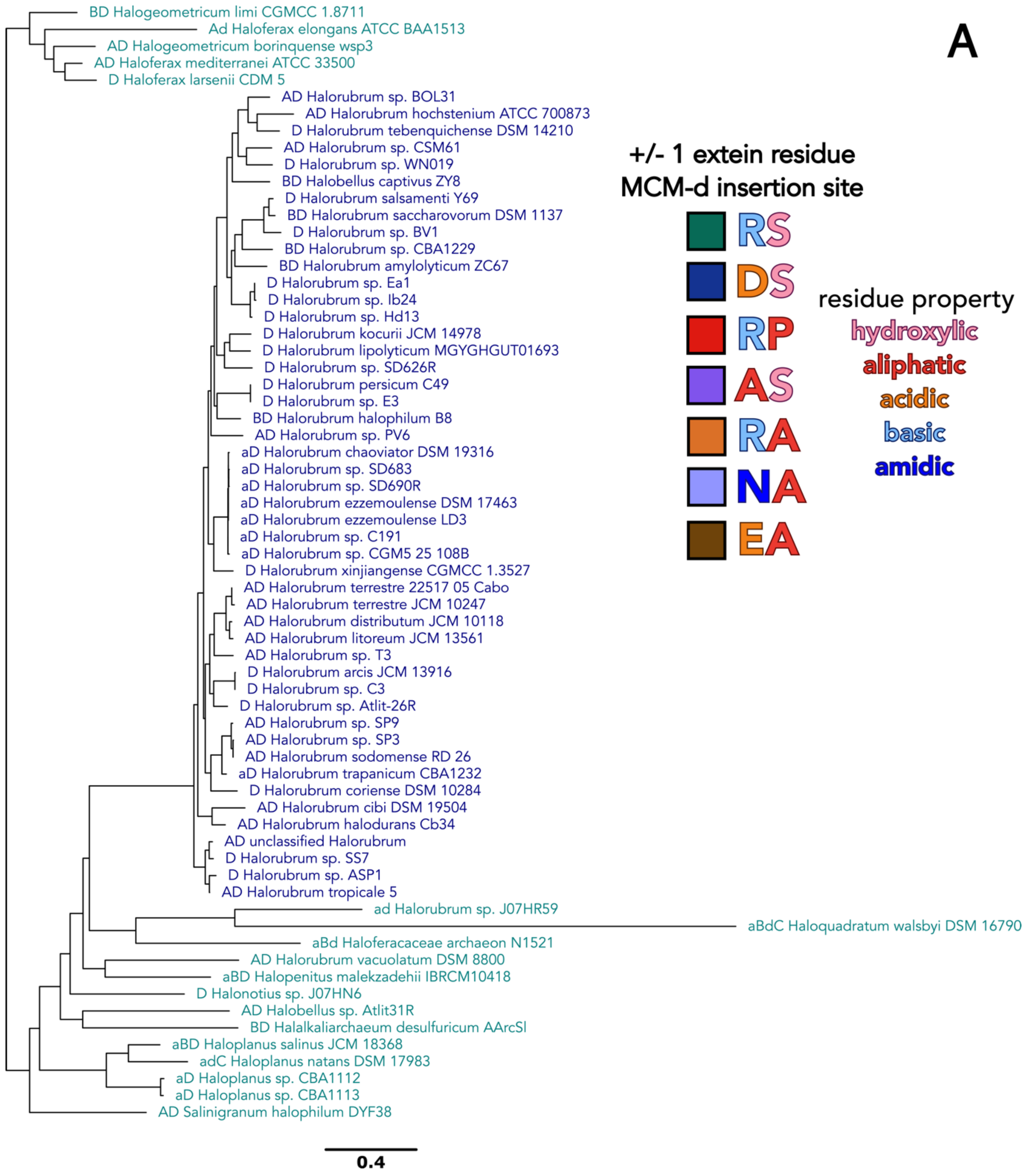
A
BD Halogeometricum limi CGMCC 1.8711
Ad Haloferax elongans ATCC BAA1513
AD Halogeometricum borinquense wsp3
AD Haloferax mediterranei ATCC 33500
D Haloferax larsenii CDM 5
AD Halorubrum sp. BOL31
AD Halorubrum hochstenium ATCC 700873
D Halorubrum tebenquichense DSM 14210
AD Halorubrum sp. CSM61
D Halorubrum sp. WN019
BD Halobellus captivus ZY8
D Halorubrum salsamenti Y69
BD Halorubrum saccharovorum DSM 1137
D Halorubrum sp. BV1
BD Halorubrum sp. CBA1229
BD Halorubrum amylolyticum ZC67
D Halorubrum sp. Ea1
D Halorubrum sp. Ib24
D Halorubrum sp. Hd13
D Halorubrum kocurii JCM 14978
D Halorubrum lipolyticum MGYGHGUT01693
D Halorubrum sp. SD626R
D Halorubrum persicum C49
D Halorubrum sp. E3
BD Halorubrum halophilum B8
AD Halorubrum sp. PV6
aD Halorubrum chaoviator DSM 19316
aD Halorubrum sp. SD683
aD Halorubrum sp. SD690R
aD Halorubrum ezzemoulense DSM 17463
aD Halorubrum ezzemoulense LD3
aD Halorubrum sp. C191
aD Halorubrum sp. CGM5 25 108B
D Halorubrum xinjiangense CGMCC 1.3527
AD Halorubrum terrestre 22517 05 Cabo
AD Halorubrum terrestre JCM 10247
AD Halorubrum distributum JCM 10118
AD Halorubrum litoreum JCM 13561
AD Halorubrum sp. T3
D Halorubrum arcis JCM 13916
D Halorubrum sp. C3
D Halorubrum sp. Atlit-26R
AD Halorubrum sp. SP9
AD Halorubrum sp. SP3
AD Halorubrum sodomense RD 26
aD Halorubrum trapanicum CBA1232
D Halorubrum coriense DSM 10284
AD Halorubrum cibi DSM 19504
AD Halorubrum halodurans Cb34
AD unclassified Halorubrum
D Halorubrum sp. SS7
D Halorubrum sp. ASP1
AD Halorubrum tropicale 5
ad Halorubrum sp. J07HR59
aBdC Haloquadratum walsbyi DSM 16790
aBd Haloferacaceae archaeon N1521
AD Halorubrum vacuolatum DSM 8800
aBD Halopenitus malekzadehii IBRCM10418
D Halonotius sp. J07HN6
AD Halobellus sp. Atlit31R
BD Halalkaliarchaeum desulfuricum AArcSl
aBD Haloplanus salinus JCM 18368
adC Haloplanus natans DSM 17983
aD Haloplanus sp. CBA1112
aD Haloplanus sp. CBA1113
AD Salinigranum halophilum DYF38
0.4
+/- 1 extein residue
MCM-d insertion site
RS
DS
RP
AS
RA
NA
EA
residue property
hydroxylic
aliphatic
acidic
basic
amidic

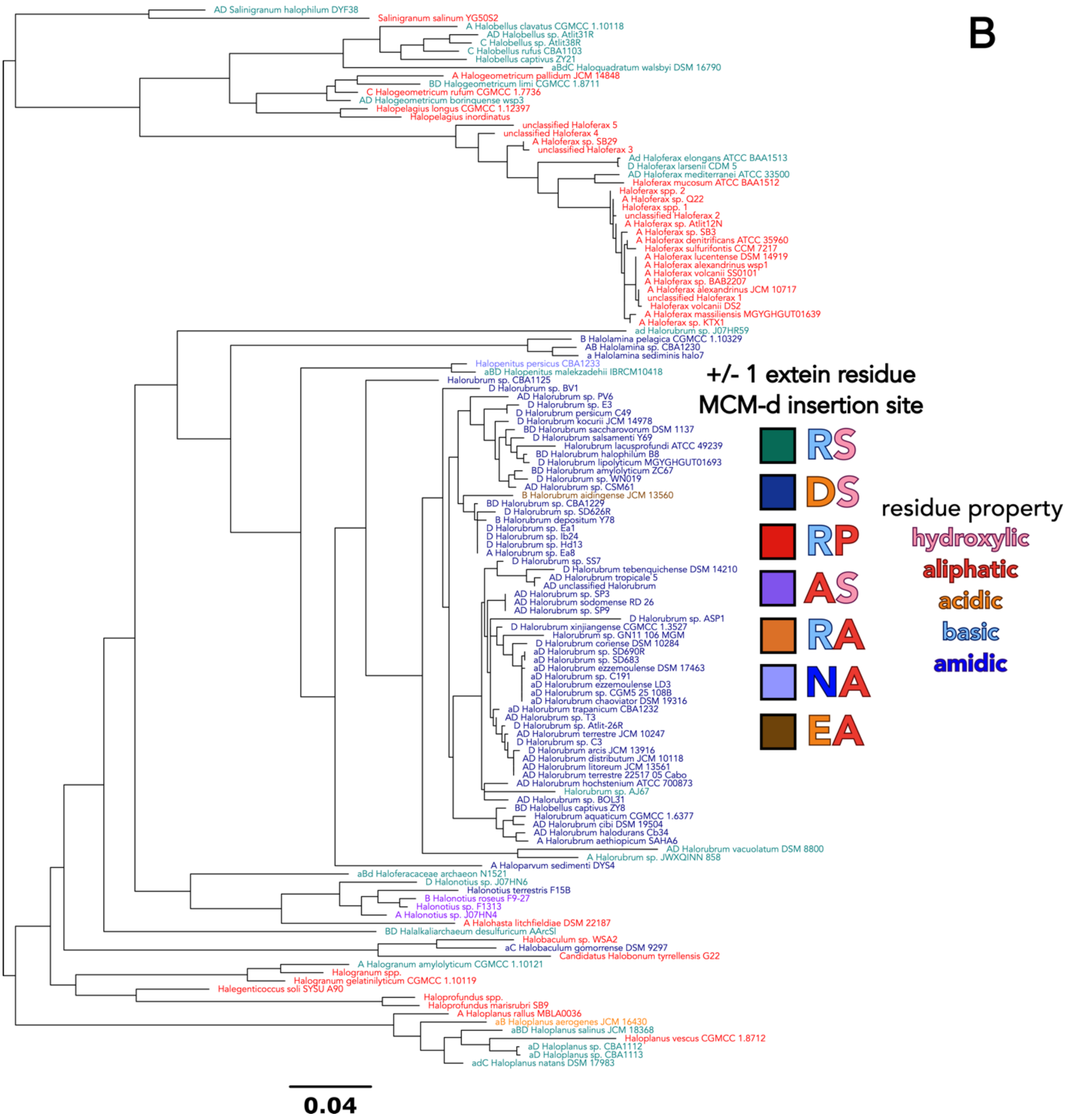

Fig. S3. Phylogeny of MCM-d inteins (panel A) and MCM extein phylogeny (panel B) colored according to MCM-d insertion site type. For an example: the red coloring indicates that the intein insertion site is between an Arginine (R) and Proline (P). Three residues on the N and C-terminal sides of the MCM-d insertion site were extracted from the extein multiple sequence alignment for all 129 entries and used to inform coloring. There are 7 distinct insertion site patterns, with RP, DS, and RS being the most widespread. The prefix to the name indicates the size of the intein: uppercase and lowercase letters denote intein sizes above and below 400 amino acids for MCM-a and 600 amino acids for MCM-d. See Supplementary Dataset S2 for the tree files in computer readable Newick format and Supplementary Dataset S1 for the partitioned alignment of the MCM proteins in nexus format.

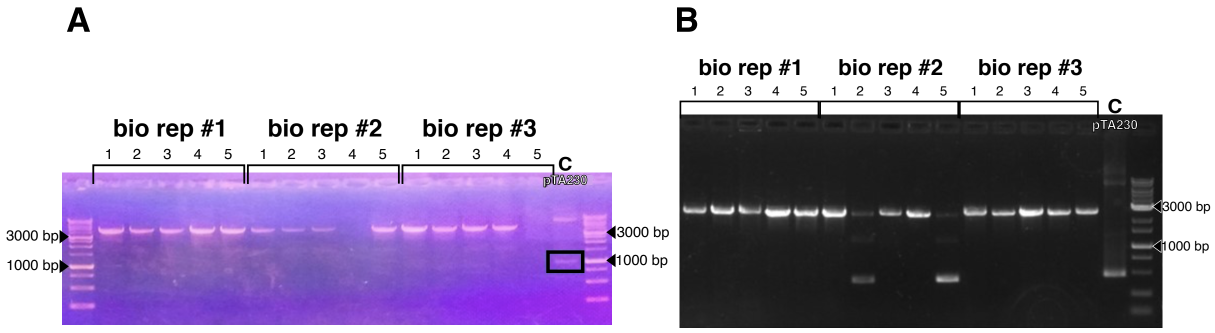

Fig. S4: A. PCR screening of WR646 colonies positive for the "mext-a target plasmid" using primers IS542+IS543. The elongated and invaded products are 4453bp, whereas nonintegrated products are 1060bp. B. PCR screening of WR646 colonies positive for the "mext-d target plasmid" using primers IS687+IS688. The elongated products are 2407bp, whereas nonintegrated products are 472bp.

**mext-a**	1	GTCCCTGGTACAGGGAAGTCTCAGATGCTGTCATA	One mismatch
	2	X	Deletion of the extein site
	3	GTCCCTGGTACAGGGAAGTCTCAGATGCTGTCATA	One mismatch
	4	GTCCCTGGTACAGGGAAGTCTCAGATGCTGTCATA	One mismatch
	5	X	Deletion of the extein site
**vext-a**	1	GACCCCGGAACTGACAAATCGCAGATGTTATCATA	One mismatch
	2	GACCCCGGAACTGGCAAATCGCAGATGTTATCATA	
	3	GACCCCGGAACTGGCAAATCGCAGATGTTATCATA	
	4	GACCCCGGAACTGGCAAATCGCAGATATTATCATA	One mismatch
	5	GACCCCGGAACTGGCAAATCGCAGATGTTATCATA	

Fig. S5: Sequence of the target site of each of the plasmids: mext-a (*mediterranei*-derived) and vext-a (*volcanii*-derived), in five randomly picked colonies of *Hfx. mediterranei* after transformation. X marks a deletion of the extein site.

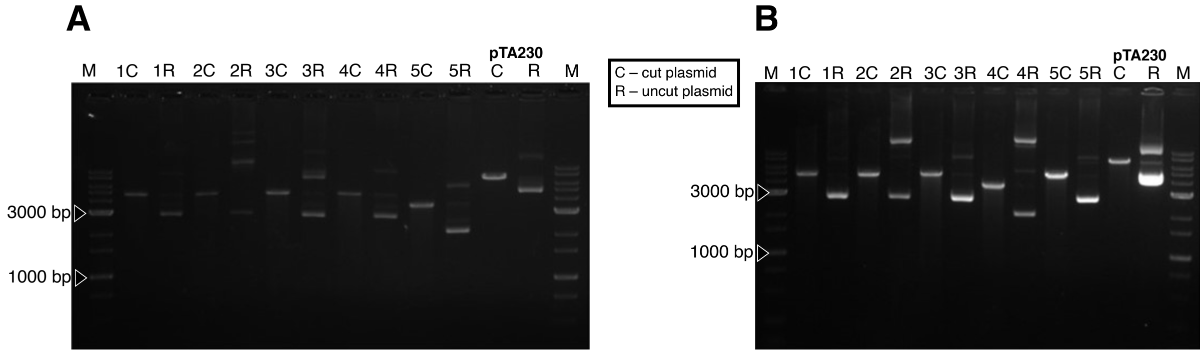

Fig. S6: A. Agarose gel representing mext-d short plasmids extracted from WR646 colonies. B. Agarose gel of vext-d short plasmid extracted from WR646 colonies. The colony number is written at the top of the gel. "C" represents linear plasmids, while "R" represents circular plasmids. As control, pTA230 was used in its linear and circular forms.

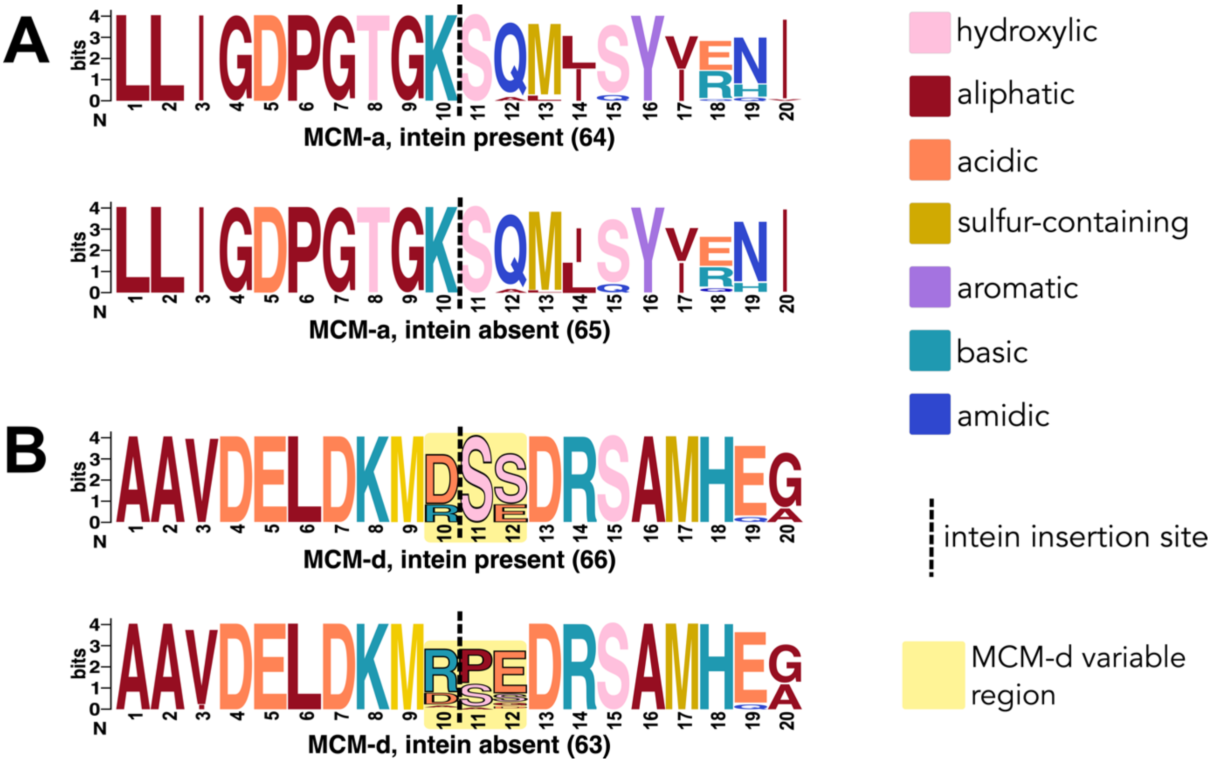

Fig. S7: Representation of amino acid proportions of the extein residues twenty positions upstream and downstream of the intein insertion site. Intein presence includes both full and mini inteins.
A. Representation of the 64 MCM-a sites with an intein present and the 65 intein-free MCM-a sites.
B. Representation of the 66 MCM-d sites with an intein present and the 63 intein-free MCM-d sites.
For MCM-a, the insertion site is conserved regardless of presence/absence. In MCM-d the +1 residue varies based on invasion.

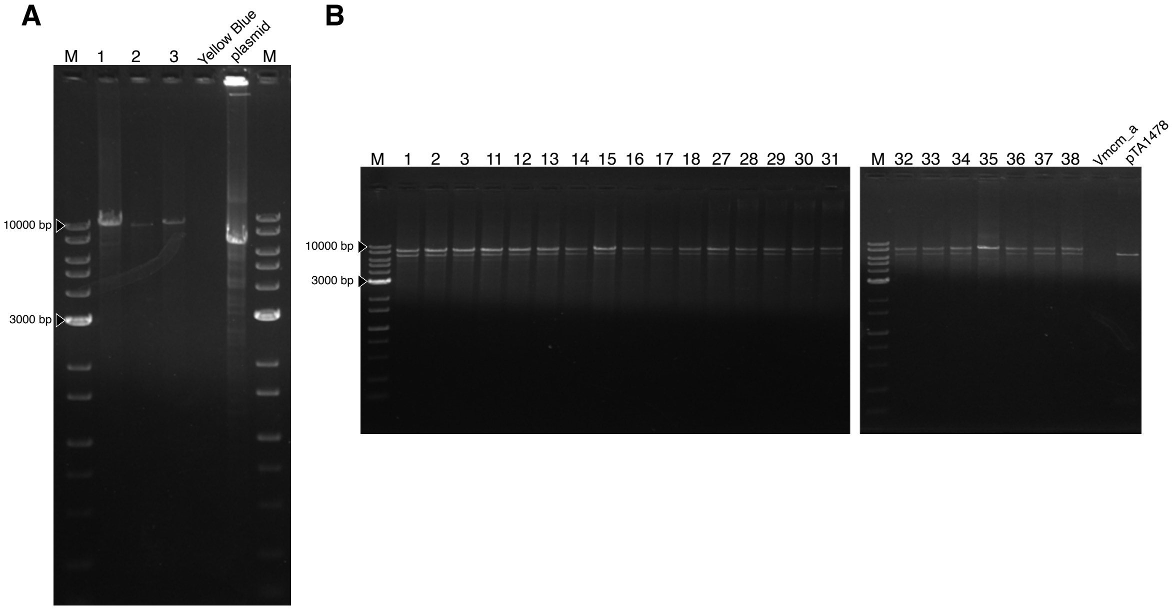

Fig. S8: A. PCR screening of pTA1478 positive *Hfx. volcanii Vmcm_a+d* colonies using GM25 and GM26 primers specific for sites surrounding the intein integration region on the plasmid. The elongated invaded products are 10,000 bp, whereas nonintegrated products are 6,500bp.
B. PCR screening of pTA1478 positive *Hfx. volcanii Vmcm_a* colonies using GM25 and GM26 primers specific for sites surrounding the intein integration region on the plasmid. The elongated invaded products are 8,000 bp, whereas nonintegrated products are 6,500bp.

vector pTA230 on its multiple cloning site):
mext-a target plasmid after invasion (primers used for sequencing are from the plasmid)
MCM-d intein
MCM-a intein
Mext-a target site
mcm helicase gene

>Colony1_Forward
NNNNNNNNGNNNNNNNNNANCCNGTCTTCGACTACTACATGGAGGGCATCTCGCTCGCTATCGAGGACGAGGAGTTCGAGG
ATATGGAGATTACCGACGAGGACGTGGCTCAAATCGTCGAACTCTCCAACAAGCCCGACATCTACGAGGAGATGACCGAC
TCCGTGGCCCCGGCCATCTACGGCTACGACCAGGAGAAACTCGCCATGATTCTCCAACTCTTCTCGGGTGTCACGAAGCA
CCTTCCCGACGGGTCTCGGATTCGCGGTGACCTGCACATGCTTCTGATAGGGGACCCTGGTACAGGGAAGTGCGTCACCG
GCGACACGCGGGTAACGCTTGCCGATGGGCGCGACGTACCGATTCGAGAGTTGGTCGAGTCGAATCTCGACGACCCGAAA
CCAATCGACGACGGTGTGTGGGACAGCGCGAACTTCGAGGTCCCATCTCTCGCCCCCGACGGGACGTTGACCTCCCGGCA
GGCGACGAAAGTTTGGAAGCGCGAAGCGCCGGACCACTTGCTGCGCATCCGAACGAACACCGGACGGGAACTCACAGCGA
CCCCATCTCATCCGCTTTTCATCCGCGACGAGGGGATGACTGCTGCCGTTCGTTCCGACGAATTGACCGAGGGAGAATTC
GTCGCAGCACCGCGCTCACTCCCAATGACCGGGACCGACGACCTTGCGGTCGAATTCCGCCGGTCGAAGTCGTACAACGC
GGTTCGTCTGGACCTGCCGGACGAGTGGACGCCGTGGCTTGCTCGGCTCGTTGGCTACATCGTTGCAGANGGGTACGTCG
AACAGCGCGACGATGCCACTGGGTTCGTCTCTGTGACNAACAACNACCGCGAGGNGTTGGACGACGNAGCTGACGCATNT
CGACCGANTGGGGACTGANCTATTTTCGAGCGGTCGCGCNNNGNATGGGNAAATGNGCTCGTGNAGNATTATCGGGTGGG
CTCTGGCNNGTNTCNTCAGCNTTNNNNCGGNNGANCCGNNNTNNCTTTGNCGNNCTNNCAANGCNTCNNNANGGNNAANN
NNANNNCCNCTGANNNNNNNTGGNNNTNNNNANNNTTCGNNAANNCNGANGACGAN

>Colony1_reverse
NNNNNNGGNGGNNCNGCCGTNNTNNANTAGTGGATCCCCCGGGCTAGAGCTGATTTTGACGTTGCCGGAATCGGTATCGA
CACACCCTTCGCTATCAGCCATCGCGCGCACGAACGCCGCTTTCGCCGCCAGCGAAGCCTCCGAAACGGCAGCCGGGAAC
GCTTTTCCATCATAGATTTCGAGGTTCATCCCGGCATCAAGTACCGCGTCAGCGTACTCACGGCCCGGTAGCCGTACGGT
CTCGACGCCGTCGTCACGCTGTTCAGACGGGGGGCGAACCGGTTCCACACCGAAGGTGTCGCGACAGACCTGCTCGAAGT
CGGCGAGCAGTTCCTCCTCCTTGTTGGTGAATCGGATACCGTAGGAGCCCGCCTCACGGTTGTAATACACGTTCCCGTCG
CCGGAAAGGTAGCCGAGTATGGCACCAAACGCAGGGGAGAGTTCGTCTTCGGTGGCAGTGATCGTCGCCTCAGCGGTCGA
TTCGGCAGTTAGGACGCCCCCATCCGTCGCTCGGGGAGAGAGGTGACGCGGGACGTAGACCCAATCGCCGGGGGAGACAT
CTGCTGCGGGCTTCTCTACACGCTCACCGTTTTCGAACGTGATGAACGGGTGGTCGTGAATCGCGGTGAGTTGCTCACCG
GACTGCAACGTTATCTGTGTCAGTTCATCCGGTGCATCGTATTCATGGATAGCCGTGACCGGACGAGTCACCNATCTCCC
GTCCTCAGTCATCGTCCGCGCTTCTGCGGCGACGTTCCAAATCNNNNGAGNGTTCNGTANTTCCTCGGTNNNANNCCNCA
CATGCAGCGTCGTGTGC

>Colony2_Forward
NNNNNNNNGNNNNNNNNNANCCNGTCTTCGACTACTACATGGAGGGCATCTCGCTCGCTATCGAGGACGAGGAGTTCGAGG
ATATGGAGATTACCGACGAGGACGTGGCTCAAATCGTCGAACTCTCCAACAAGCCCGACATCTACGAGGAGATGACCGAC
TCCGTGGCCCCGGCCATCTACGGCTACGACCAGGAGAAACTCGCCATGATTCTCCAACTCTTCTCGGGTGTCACGAAGCA
CCTTCCCGACGGGTCTCGGATTCGCGGTGACCTGCACATGCTTCTGATAGGGGACCCTGGTACAGGGAAGTGCGTCACCG
GCGACACGCGGGTAACGCTTGCCGATGGGCGCGACGTACCGATTCGAGAGTTGGTCGAGTCGAATCTCGACGACCCGAAA
CCAATCGACGACGGTGTGTGGGACAGCGCGAACTTCGAGGTCCCATCTCTCGCCCCCGACGGGACGTTGACCTCCCGGCA
GGCGACGAAAGTTTGGAAGCGCGAAGCGCCGGACCACTTGCTGCGCATCCGAACGAACACCGGACGGGAACTCACAGCGA
CCCCATCTCATCCGCTTTTCATCCGCGACGAGGGGATGACTGCTGCCGTTCGTTCCGACGAATTGACCGAGGGAGAATTC
GTCGCAGCACCGCGCTCACTCCCAATGACCGGGACCGACGACCTTGCGGTCGAATTCCGCCGGTCGAAGTCGTACAACGC
GGTTCGTCTGGACCTGCCGGACGAGTGGACGCCGTGGCTTGCTCGGCTCGTTGGCTACATCGTTGCAGANGGGTACGTCG
AACAGCGCGACGATGCCACTGGGTTCGTCTCTGTGACNAACAACNACCGCGAGGNGTTGGACGACGNAGCTGACGCATNT
CGACCGANTGGGGACTGANCTATTTTCGAGCGGTCGCGCNNNGNATGGGNAAATGNGCTCGTGNAGNATTATCGGGTGGG
CTCTGGCNNGTNTCNTCAGCNTTNNNNCGGNNGANCCGNNNTNNCTTTGNCGNNCTNNCAANGCNTCNNNANGGNNAANN
NNANNNCCNCTGANNNNNNNTGGNNNTNNNNANNNTTCGNNAANNCNGANGACGAN

>Colony2_ reverse
NNNNNNNNNGNNNNNNNNNCCGCTCTAGAACTAGTGGATCCCCCGGGCTAGAGCTGATTTTGACGTTGCCGGAATCGGTAT
CGACACACCCTTCGCTATCAGCCATCGCGCGCACGAACGCCGCTTTCGCCGCCAGCGAAGCCTCCGAAACGGCAGCCGGG
AACGCTTTTCCATCATAGATTTCGAGGTTCATCCCGGCATCAAGTACCGCGTCAGCGTACTCACGGCCCGGTAGCCGTAC
GGTCTCGACGCCGTCGTCACGCTGTTCAGACGGGGGGCGAACCGGTTCCACACCGAAGGTGTCGCGACAGACCTGCTCGA
AGTCGGCGAGCAGTTCCTCCTCCTTGTTGGTGAATCGGATACCGTAGGAGCCCGCCTCACGGTTGTAATACACGTTCCCG
TCGCCGGAAAGGTAGCCGAGTATGGCACCAAACGCAGGGGAGAGTTCGTCTTCGGTGGCAGTGATCGTCGCCTCAGCGGT
CGATTCGGCAGTTAGGACGCCCCCATCCGTCGCTCGGGGAGAGAGGTGACACGGGACGTAGACCCAATCGCCGGGGGAGA
CATCTGCTGCGGGCTTCTCTACACGCTCACCGTTTTCGAACGTGATGAACGGGTGGTCGTGAATCGCGGTGAGTTGCTCA
CCGGACTGCAACGTTATCTGTGTCAGTTCATCCGGTGCATCGTATTCATGGATAGCCGTGACCGGTCGAGTCACCAATCT
CCCGTCCTCAGTCATCGTCCACGCTTCTGCGTCGACGTTCCGAATCGTTCGACCGTTCGGTAATTCCTCGATAGACCCCT
CACATGCAGCGTCGTGTGCNAAGGTCTCTGATGCGTTTGATTCCCCCGTCAGCGAGGTGGACGAGGGAGTCGCCAGTCAC
ACACCGCATCTTGTCAAGCTCGTCGACAGCCGCAATCCCCTTGTCCGCAAGCACGAGGGCACCCGCTTCGAGCGTCCACT
GCTGGGCCGNCNNCGAAATCGTCGCGAANCGCCGNANCNGNGAGCCCTGCTGANGANNNNNNCNNNNNGTAGACNGANNN
NGCGNGATATTCNAATNNNTGANNNCATCNTGNNNTTANGCNNNNCNANNNNNNNNNANNNNANNNTGNGGNNNNNNNTNN
ANNNNNNNNNNNNNANNNNTTTNNNNNNN

>Colony3_Forward
NNNNNNNNNNNANNNNNNNNNNCCNGTCTTCNACTACTACATGGAGGGCATCTCGCTCGCTATCGAGGACGAGGAGTTCGA
GGATATGGAGATTACCGACGAGGACGTGGCTCAAATCGTCGAACTCTCCAACAAGCCCGACATCTACGAGGAGATGACCG
ACTCCGTGGCCCCGGCCATCTACGGCTACGACCAGGAGAAACTCGCCATGATTCTCCAACTCTTCTCGGGTGTCACGAAG
CACCTTCCCGACGGGTCTCGGATTCGCGGTGACCTGCACATGCTTCTGATAGGGGACCCTGGTACAGGGAAGTGCGTCAC
CGGCGACACGCGGGTAACGCTTGCCGATGGGCGCGACGTACCGATTCGAGAGTTGGTCGAGTCGAATCTCGACGACCCGA

AACCAATCGACGACGGTGTGTGGGACAGCGCGAACTTCGAGGTCCCATCTCTCGCCCCCGACGGGACGTTGACCTCCCGG
CAGGCGACGAAAGTTTGGAAGCGCGAAGCGCCGGACCACTTGCTGCGCATCCGAACGAACACCGGACGGGAACTCACAGC
GACCCCATCTCATCCGCTTTTCATCCGCGACGAGGGGATGACTGCTGCCGTTCGTTCCGACGAATTGACCGAGGGAGAAT
TCGTCGCAGCACCGCGCTCACTCCCAATGACCGGGACCGACGACCTTGCGGTCGAATTCCGCCGGTCGAAGTCGTACAAC
GCGGTTCGTCTGGACCTGCCGGACGAGTGGACGCCGTGGGCTTGCTCGGCTCGTTGGCTACATCGGTTGCAGAGGGGTAC
GTCGAACAGCGCGACGATGCCACTGGGGTTCGTCTCTGTGACGAACAACGACCGCGANGTGTNGNGACGACGTAGCTGAC
GCATTNCGACCGATTGGGGACTGAACTATTTCCGAGCGGNCGGCCCCACNANTGGGAAATTCCGCGTCCNTGANATTTAT
CTGTGGGNTCTGGGNNNNTTTCNNNNNNGNTTCNGCTNNNANACCTCTNNNNCTTTCNNNTTCNTNNNNGGGTCCNGANN
NNCCNNNCGCNATCCCTTNNNGGGNGGGGTGGTNGATCTGCNNANNTNNNNNNNANATNNNGNATGANNNNNTGT

>Colony3_ reverse
NNNNNNNNNGNGNNCNNNCNCNCTAGAACTAGTGGATCCCCCGGGCTAGAGCTGATTTTGACGTTGCCGGAATCGGTATC
GACACACCCTTCGCTATCAGCCATCGCGCGCACGAACGCCGCTTTCGCCGCCAGCGAAGCCTCCGAAACGGCAGCCGGGA
ACGCTTTTCCATCATAGATTTCGAGGTTCATCCCGGCATCAAGTACCGCGTCAGCGTACTCACGGCCCGGTAGCCGTACG
GTCTCGACGCCGTCGTCACGCTGTTCAGACGGGGGGCGAACCGGTTCCACACCGAAGGTGTCGCGACAGACCTGCTCGAA
GTCGGCGAGCAGTTCCTCCTCCTTGTTGGTGAATCGGATACCGTAGGAGCCCGCCTCACGGTTGTAATACACGTTCCCGT
CGCCGGAAAGGTAGCCGAGTATGGCACCAAACGCAGGGGAGAGTTCGTCTTCGGTGGCAGTGATCGTCGCCTCAGCGGTC
GATTCGGCAGTTAGGACGCCCCCATCCGTCGCTCGGGGAGAGAGGTGACACGGGACGTAGACCCAATCGCCGGGGGAGAC
ATCTGCTGCGGGCTTCTCTACACGCTCACCGTTTTCGAACGTGATGAACGGGTGGTCGTGAATCGCGGTGAGTTGCTCAC
CGGACTGCAACGTTATCTGTGTCAGTTCATCCGGTGCATCGTATTCATGGATAGCCGTGACCGGTCGAGTCACCAATCTC
CCGTCCTCAGTCATCGTCCACGCTTCTGCGTCGACGTTCCGAATCGTTCGACCGTTCGGTAATTCCTCGATAGACCCCTC
ACATGCAGCGTCGTGTGCAAGGTCTCTGATGCGTTTGATTCCCCCGTCAGCGAGGTGGACGAGGGAGTCGCCAGTCACAC
ACCCGCATCTTGTCANGCTCGTCNNACAGCCNCAATTCCCCNTNGTCCGNNNGNNCGAAGGGCNCCCNGCTTCGAGCGTC
CCACTTGCTGGGCCGNCANCCNNAAATCGTNNNGANCGNCNNANCGGTGANCCCNTGCTGACNANNAANCCNTTNCCTGG
ANGNGNNGACNGANNNNNNNNNNNGATNNTNNNNANNATNGACNGCCANNNNGNNNTCTNTNNNNNNNNNNNNNNCCNNNNN
NGNANACNNNNNNNNNNNNNN

mext-d target plasmid after invasion (primers used for sequencing are from the plasmid vector pTA230 on its multiple cloning site):
MCM-d intein
MCM-a intein
Mext-a target site
mcm helicase gene
Mext-d target site

>Colony1_Forward
NNTCANNANGNGTCGACCCGCCGAAAATGGGTCTACGACCTCGAAGTCGAAGGCACCCACAGCTACGTTTCCAACGGGCT
CGTCTCGCATAATTCTCAGATGCTGTCATATATTCGAAATATCGCGCCGCGTTCTGTCTACACCTCCGGGAAGGGTTCTT
CGTCAGCAGGGCTCACCGCTGCGGCGGTTCGCGACGATTTCGGTGACGGCCAGCAGTGGACGCTCGAAGCGGGTGCCCTC
GTGCTTGCGGACAAGGGGATTGCGGCTGTCGACGAGCTTGACAAGATGCGGTGTGTGACTGGCGACTCCCTCGTCCACCT
CGCTGACGGGGGAATCAAACGCATCAGAGACCTTGCACACGACGCTGCATGTGAGGGGTCTATCGAGGAATTACCGAACG
GTCGAACGATTCGGAACGTCGACGCAGAAGCGTGGACGATGACTGAGGACGGGAGATTGGTGACTCGACCGGTCACGGCT
ATCCATGAATACGATGCACCGGATGAACTGACACAGATAACGTTGCAGTCCGGTGAGCAACTCACCGCGATTCACGACCA
CCCGTTCATCACGTTCGAAAACGGTGAGCGTGTAGAGAAGCCCGCAGCAGATGTCTCCCCCGGCGATTGGGTCTACGTCC
CGTGTCACCTCTCTCCCCGAGCGACGGATGGGGGCGTCCTAACTGCCGAATCGACCGCTGAGGCGACGATCACTGCCACC
GAAGACGAACTCTCCCCTGCGTTTGGTGCCATACTCGGCTACCTTTCCGGCGACGGGAACGTGTATTACAACCGTGAGGC
GGGCTCCTACGGTATCCGATTCACCAACAAGGAGGAGGAACTGCTCGCCGACTTCGAGCAGGTCTGTCGCGACACCTTCG
GTGTGGAACCGGGTTCGCCCCCCGTCTGAACAGCGTGACGACGGCGTCGAGACCGTACGGCTACCGGGCCGTGAGTACGC
TGACGCCGGTACTTTGATGCCGGGATGAACCTCGAAATCTATGATGGGAAAAGCGTTCCCCGGCCTGCCCGTTCCGGAGG
CTTCCGCCTGGCCGCCGAAAGCCGCGTTCGTGGCCGCCGCCGATGGCCTGAATAGCNAGGGTGGTGNTCCGANNNCGATT
NNGNACGTCNANTACAGGCTNTNNGGCTACCGGAACTGCTGCTCNNNTCAAGGNNNNNNNTTNGNGTTCNNGGGNGGTAC
ACGCAG

>Colony1_Reverse
GCGACCCCGTCCTTCGGGTTTGCCGCGCCCGAGGAGCGAACAACGCGATTTGAGCGTCGCGTTGATACCCGCCTTGGAGA
CCGAAATCTGCTGTTGTTCGAGCGCTTCGTGCATAGCCGAACGGTCTTCGGAGTTGTGCACGACCATTCCATTGGCGACG
AAGTTGTGCGTTCCCTCGACAGTCAGGTCGTACACCTTTGGCTTCGAACGGGACCCGATGGCATCGAGAAGGCGTCGAGC
ATCCGAACGAATGTCGGAGAAGCGCGCGTTTGCCACCTCAACCACGTCAGTAAACCGGTCCGTCTCGACGACGCCGTTGA
ACCACCGCGAGACGGTCGAGCCAGCAACATCGAGGTCGCGCGCGATGTCAGCGAAAGAGACACCGTGGCGTTCGAGTTCG
GAACGAAGTTCGCTCCAGTCGTCTGCACGCGGTTCGTGATCTAACAGCTGCTGTGCGTGTTCGACGGCCGCATCACCCTC
GACGCCGAGGAGGTCCGCAAGCTCGTGTTTTAGTAGCCGAATTCGGTCGTCGGCATCGCTCTCGTCGACTGTTTCGACAG
CCCTTCACGCGTCTCCACTTGACATCACCCCGAACGAGTCGTCGGATCGATTCGAGGTCCCGTGTCAGCGACTGATTCAA
CGATTTCACGGAGCCCGGCCTCTCGTGACTGCTCTGAGTTCGTCGTCCGTACCCCAGAGCCGAGAGATCTGTTGCTGGCT
GAACGAAGTTCCACTGCCAACTCAGACTGAGAGACGTGGTCACGTTCTTTAACCAATCGAAATCGTCGCCCCAGTCACAC
GCGTTCAGAGAGTCTGCGAGACGTCCTGACTCGGCCCTGTGACATTCCGACTCTCAAACGCGTCGAGAACCGTTCGAGCA
CATGAGAACGAGATGTTCGCGTCCTCGTTCTCGCACGTTGCAGTAGGTCGAATCGGACGGAGTCCCGCATTCGGAGACGT
GGAAGGCCGTAGCGACTTCGCGGAGCGACCGGATAGGTACCCCCTCCAACAGGTTCGGGTATACACGTTGGAGAATTCGT
CGGTCAACCCGANACGGACTCACAAGACCTTGTCAGGCGCCGAATTGTTGGCGTCTCCAGGTCGTGAATTCGGATATGNC
GGTTTGAACCCNCCCGNNGGCNNNNNTTGCATGNCTGCTACACGCGCANGTTAACGCTA

>Colony2_Forward
NTCACNNNGCGTCGANCCGCCGAAAATGGGTCTACGACCTCGAAGTCGAAGGCACCCACAGCTACGTTTCCAACGGGCTC
GTCTCGCATAATTCTCAGATGCTGTCATATATTCGAAATATCGCGCCGCGTTCTGTCTACACCTCCGGGAAGGGTTCTTC
GTCAGCAGGGCTCACCGCTGCGGCGGTTCGCGACGATTTCGGTGACGGCCAGCAGTGGACGCTCGAAGCGGGTGCCCTCG
TGCTTGCGGACAAGGGGATTGCGGCTGTCGACGAGCTTGACAAGATGCGGTGTGTGACTGGCGACTCCCTCGTCCACCTC

GCTGACGGGGGAATCAAACGCATCAGAGACCTTGCACACGACGCTGCATGTGAGGGGTCTATCGAGGAATTACCGAACGG
TCGAACGATTCGGAACGTCGACGCAGAAGCGTGGACGATGACTGAGGACGGGAGATTGGTGACTCGACCGGTCACGGCTA
TCCATGAATACGATGCACCGGATGAACTGACACAGATAACGTTGCAGTCCGGTGAGCAACTCACCGCGATTCACGACCAC
CCGTTCATCACGTTCGAAAACGGTGAGCGTGTAGAGAAGCCCGCAGCAGATGTCTCCCCCGGCGATTGGGTCTACGTCCC
GTGTCACCTCTCTCCCCGAGCGACGGATGGGGGCGTCCTAACTGCCGAATCGACCGCTGAGGCGACGATCACTGCCACCG
AAGACGAACTCTCCCCTGCGTTTGGTGCCATACTCGGCTACCTTTCCGGCGACGGGAACGTGTATTACAACCGTGAGGCG
GGCTCCTACGGTATCCGATTCACCAACAAGGAGGAGGAACTGCTCGCCGACTTCGAGCAGGTCTGTCGCGACACCTTCGG
TGTGGAACCGGTTCGCCCCCCGTCTGAACAGCGTGACGACGGCGTCGAGACCGTACGGCTACCGGGCCCGTGAGTACGCT
GACGCGTACTTGATGCCGGNTGAACNTCGAAATCTATGATGGAAAAGCGTCCCGCTGCGTTCGNNGCTTCGCTGNNGNGA
ANCGNGGTTCGNGCGCGCGATGCTGAATAGCGNNNNGTGTCGGATACCGANCNNNACGTTCAATCAGCCTNNTCCAGGNT
ACGANTGNNNNTCNNNNNCNNAGNTNNCCCTTNGAGTTNGCGNGTACNGANNCGC

>Colony2_Reverse
GCGACGACGTATTCGGGTTTGCCGCGCCGAGGAGCGAACAACGCGATTTGAGCGTCGCGTTGATACCCGCCTTGGAGACC
GAAATCTGCTGTTGTTCGAGCGCTTCGTGCATAGCCGAACGGTCTTCGGAGTTGTGCACGACCATTCCATTGGCGACGAA
GTTGTGCGTTCCCTCGACAGTCAGGTCGTACACCTTTGGCTTCGAACGGGACCCGATGGCATCGAGAAGGCGTCGAGCAT
CCGAACGAATGTCGGAGAAGCGCGCGTTTGCCACCTCAACCACGTCAGTAAACCGGTCCGTCTCGACGACGCCGTTGAAC
CACCGCGAGACGGTCGAGCCAGCAACATCGAGGTCGCGCGCGATGTCAGCGAAAGAGACACCGTGGCGTTCGAGTTCGGA
ACGAAGTTCGCTCCAGTCGTCTGCACGCGGTTCGTGATCTAACAGCTGCTGTGCGTGTTCGACGGCCGCATCACCCTCGA
CGCCGAGGAGGTCCGCAAGCTCGTGTTTTAGTAGCCGAATTCGGTCGTCGGCATCGCTCTCGTCGACTGTTTCGACAGCC
TTCACGCGTCTCCACTTGACATCACCCCGAACGAGTCGTCGGATCGATTCGAGGTCCGTGTCAGCGACTGATTCAACGAT
TTCACGGAGCCGGCCTCTCGTGACTGCTCTGAGTTCGTCGTCCGTACCCCAGAGCCGAGAGATCTGTTGCTGGCTGAACG
AAGTTCCCACTGCCAACTCAGACTGAGAGACGTGGTAACGTTCTTTAACAATCGAAATCGTCGCCCAGTCACACGGTTCA
GAGAGTTGCGAGACGTCTGACTCGCCCTGTGACATTCGACTCTCAAACGCGTCGAGAACCGTTCGAGCACATGAGAACGA
GATGTTCGCGTCCTCGTTCTCGAAGTTGCAGTAGGTCGAATCGACGAGTCCGCATTCGGAGACGTGAAGCCGTAGCGACT
CGCGGAGCGACGATAGTACCCCTCCACAGTTCGGAATCACGTCGAGAATCGTTCGTCACCCGAGACGAANNNACAAGACC
TCGTCAAGCGCCGATTGTTTGCGTTCCAGTTGTGAAATCNATNTGNNTGAANNCNGAAGNNNGTCTGCTGCTGTNACCGC
AGTACGTAACAAAGATTCCNNNNNNAANNNNNNNGNTGCTTGAATNTGGCGTNCGNNNNCGCCCGAANTTCNACNAN

>Colony3_Forward
NCNCNNGACGCGTCGANCCGCCGAAAATGGGTCTACGACCTCGAAGTCGAAGGCACCCACAGCTACGTTTCCAACGGGCT
CGTCTCGCATAATTCTCAGATGCTGTCATATATTCGAAATATCGCGCCGCGTTCTGTCTACACCTCCGGGAAGGGTTCTT
CGTCAGCAGGGCTCACCGCTGCGGCGGTTCGCGACGATTTCGGTGACGGCCAGCAGTGGACGCTCGAAGCGGGTGCCCTC
GTGCTTGCGGACAAGGGGATTGCGGCTGTCGACGAGCTTGACAAGATGCGGTGTGTGACTGGCGACTCCCTCGTCCACCT
CGCTGACGGGGGAATCAAACGCATCAGAGACCTTGCACACGACGCTGCATGTGAGGGGTCTATCGAGGAATTACCGAACG
GTCGAACGATTCGGAACGTCGACGCAGAAGCGTGGACGATGACTGAGGACGGGAGATTGGTGACTCGACCGGTCACGGCT
ATCCATGAATACGATGCACCGGATGAACTGACACAGATAACGTTGCAGTCCGGTGAGCAACTCACCGCGATTCACGACCA
CCCGTTCATCACGTTCGAAAACGGTGAGCGTGTAGAGAAGCCCGCAGCAGATGTCTCCCCCGGCGATTGGGTCTACGTCC
CGTGTCACCTCTCTCCCCGAGCGACGGATGGGGGCGTCCTAACTGCCGAATCGACCGCTGAGGCGACGATCACTGCCACC
GAAGACGAACTCTCCCCTGCGTTTGGTGCCATACTCGGCTACCTTTCCGGCGACGGGAACGTGTATTACAACCGTGAGGC
GGGCTCCTACGGTATCCGATTCACCAACAAGGAGGAGGAACTGCTCGCCGACTTCGAGCAGGTCTGTCGCGACACCTTCG
GTGTGGAACCGGTTCGCCCCCCGTCTGAACAGCGTGACGACGGCGTCGAGACCGTACGGCTACCGGGCCCGTGAGTACGC
TGACGCGGTACTTTGATGCCGGGNTGAACCTCGAAATCTATGATGGAAAAGCGTTCCCNNTGCCGTTTCGGAAGCTTCGC
TGGCGNGAAAGCGCGTTTCGTGCGCCGCGATGGCTGAATAGCGNAAGGNGGTGTCGATACCGAATTNNNNACGTTCAAAA
ATANNNAGNCTNNTCNNGGNTACGANTGCTGCNNGANNNAAGAANNNNCTCNCTCTTTGGANGATTNNGNNNGN

>Colony3_Reverse
GCGACGACGTATTCGGGTTTGCCGCGCCGAGGAGCGAACAACGCGATTTGAGCGTCGCGTTGATACCCGCCTTGGAGACC
GAAATCTGCTGTTGTTCGAGCGCTTCGTGCATAGCCGAACGGTCTTCGGAGTTGTGCACGACCATTCCATTGGCGACGAA
GTTGTGCGTTCCCTCGACAGTCAGGTCGTACACCTTTGGCTTCGAACGGGACCCGATGGCATCGAGAAGGCGTCGAGCAT
CCGAACGAATGTCGGAGAAGCGCGCGTTTGCCACCTCAACCACGTCAGTAAACCGGTCCGTCTCGACGACGCCGTTGAAC
CACCGCGAGACGGTCGAGCCAGCAACATCGAGGTCGCGCGCGATGTCAGCGAAAGAGACACCGTGGCGTTCGAGTTCGGA
ACGAAGTTCGCTCCAGTCGTCTGCACGCGGTTCGTGATCTAACAGCTGCTGTGCGTGTTCGACGGCCGCATCACCCTCGA
CGCCGAGGAGGTCCGCAAGCTCGTGTTTTAGTAGCCGAATTCGGTCGTCGGCATCGCTCTCGTCGACTGTTTCGACAGCC
TTCACGCGTCTCCACTTGACATCACCCCGAACGAGTCGTCGGATCGATTCGAGGTCCGTGTCAGCGACTGATTCAACGAT
TTCACGGAGCCGGCCTCTCGTGACTGCTCTGAGTTCGTCGTCCGTACCCCAGAGCCGAGAGATCTGTTGCTGGCTGAACG
AAGTTCCCACTGCCAACTCAGACTGAGAGACGTGGTAACGTTCTTTAACAATCGAAATCGTCGCCCAGTCACACGGTTCA
GAGAGTTGCGAGACGTCTGACTCGGCCTGTGACATTCGACTCTCAAACGCGTCGAGAACCGTTCGAGCACATGAGAACGA
GATGTTCGCGTCCTCGTTCTCGAAGTTGCAGTAGGTCGAATCGACGAGTCCGCATTCGGAGACGTGAAGCCGTAGCGACT
CGCGNGCGACGATAGTACCCTCCACAGTTCGGAATCACGTCGAGAATCGTTCGTCACCCGAGACGGACTCACAGACCTCG
TCAAGCGCCGATTGTTTGCGTTCCAGTGTGAATCCGATNTGCNNNGACCGNCNNNNNGTCTGCTGCTGNNACCGCAGTAC
GTACAAGNTNNNNNNNNAGCNNNNNCGGTGCTGGAATCTGCGCGTNNGTGNANGCNNNNNNNNNN

>Colony4_Forward
NNNCNCNACGCGCCGACCCGCCGAAAATGGGTCTACGACCTCGAAGTCGAAGGCACCCACAGCTACGTTTCCAACGGGCT
CGTCTCGCATAATTCTCAGATGCTGTCATATATTCGAAATATCGCGCCGCGTTCTGTCTACACCTCCGGGAAGGGTTCTT
CGTCAGCAGGGCTCACCGCTGCGGCGGTTCGCGACGATTTCGGTGACGGCCAGCAGTGGACGCTCGAAGCGGGTGCCCTC
GTGCTTGCGGACAAGGGGATTGCGGCTGTCGACGAGCTTGACAAGATGCGGTGTGTGACTGGCGACTCCCTCGTCCACCT
CGCTGACGGGGGAATCAAACGCATCAGAGACCTTGCACACGACGCTGCATGTGAGGGGTCTATCGAGGAATTACCGAACG
GTCGAACGATTCGGAACGTCGACGCAGAAGCGTGGACGATGACTGAGGACGGGAGATTGGTGACTCGACCGGTCACGGCT
ATCCATGAATACGATGCACCGGATGAACTGACACAGATAACGTTGCAGTCCGGTGAGCAACTCACCGCGATTCACGACCA
CCCGTTCATCACGTTCGAAAACGGTGAGCGTGTAGAGAAGCCCGCAGCAGATGTCTCCCCCGGCGATTGGGTCTACGTCC
CGTGTCACCTCTCTCCCCGAGCGACGGATGGGGGCGTCCTAACTGCCGAATCGACCGCTGAGGCGACGATCACTGCCACC
GAAGACGAACTCTCCCCTGCGTTTGGTGCCATACTCGGCTACCTTTCCGGCGACGGGAACGTGTATTACAACCGTGAGGC
GGGCTCCTACGGTATCCGATTCACCAACAAGGAGGAGGAACTGCTCGCCGACTTCGAGCAGGTCTGTCGCGACACCTTCG
TGTGGAACCGGTTCGCCCCCCGTCTGAACAGCGTGACGACGGCGTCGAGACCGTACGGCTACCGGGCCGTGAGTACGCTG
ACGCGGTACTTGATGCCGGGATGAACCCTCGAATCTATGATGGAAAAGCGTTCCCGGCTGCCGTTTCCGAGCTTCCGNTT

GCCGGNGAAAGCGCGTTCGTGCCGCCGCGATGCTGATAGCGAAAGGGTGTGTCGATAACCGATTCCGGCACGGTCCAAAT
NAGNNTNNTNNGCTACGGAACTGCTGNNTCNNNTACAAGAGCTNTCCNCNTNTATGGGAAGTTCCGGCGCGGNCA

>Colony4_Reverse
GCGACGACGTACTTCGGGTTTGCCGCGCCGAgGAGCGAACAACGCGATTTGAGCGTCGCGTTGATACCCGCCTTGGAGAC
CGAAATCTGCTGTTGTTCGAGCGCTTCGTGCATAGCCGAACGGTCTTCGGAGTTGTGCACGACCATTCCATTGGCGACGA
AGTTGTGCGTTCCCTCGACAGTCAGGTCGTACACCTTTGGCTTCGAACGGGACCCGATGGCATCGAGAAGGCGTCGAGCA
TCCGAACGAATGTCGGAGAAGCGCGCGTTTGCCACCTCAACCACGTCAGTAAACCGGTCCGTCTCGACGACGCCGTTGAA
CCACCGCGAGACGGTCGAGCCAGCAACATCGAGGTCGCGCGCGATGTCAGCGAAAGAGACACCGTGGCGTTCGAGTTCGG
AACGAAGTTCGCTCCAGTCGTCTGCACGCGGTTCGTGATCTAACAGCTGCTGTGCGTGTTCGACGGCCGCATCACCCTCG
ACGCCGAGGAGGTCCGCAAGCTCGTGTTTTAGTAGCCGAATTCGGTCGTCGGCATCGCTCTCGTCGACTGTTTCGACAGC
CTTCACGCGTCTCCACTTGACATCACCCCGAACGAGTCGTCGGATCGATTCGAGGTCCGTGTCAGCGACTGATTCAACGA
TTTCACGGAGCCGGCCTCTCGTGACTGCTCTGAGTTCGTCGTCCGTACCCCAGAGCCGAGAGATCTGTTGCTGGCTGAAC
GAAGTTCCCACTGCCAACTCAGACTGAGAGACGTGGTAACGTTCTTTAACAATCGAAATCGTCGCCCAGTCACACGGTTC
AGAGAGTTTGCGAGACGTCTGACTCGGCCTGTGACATTCGACTCTCAAACGCGTCGAGAACCGTTCGAGCACATGAGAAC
GAGATGTTCGCGTCCTCGTTCTCGAAGTTGCAGTAGGTCGAATCGACGAGTCCGCATTCGGAGACGTGAAGCCGTAGCGA
CTCGCGAGCGACGATAGTACCCNNCANAGTTCGGAATCACGTCGGAGAATCGTTCGGTCACCCGAGACGGACTNNCAGAC
CTCGTCAAGCGCNATTGTTTGCGTTCCCAGTNGTGATCCCGANNNGGCNNNNACCCGCCGGAGNNGTNCTGCTGNNGTNN
CNNNNGGTACGTTACAANNNNCGTNNCAGANCGTTNNGGGNNGCTGAATTNTNGCNNCGGNNNNCNCCCGNAANNNNTAG
NNN

>Colony5_Forward
NNTCACGNNGCGTCGANCCGCCGAAAATGGGTCTACGACCTCGAAGTCGAAGGCACCCACAGCTACGTTTCCAACGGGCT
CGTCTCGCATAATTCTCAGATGCTGTCATATATTCGAAATATCGCGCCGCGTTCTGTCTACACCTCCGGGAAGGGTTCTT
CGTCAGCAGGGCTCACCGCTGCGGCGGTTCGCGACGATTTCGGTGACGGCCAGCAGTGGACGCTCGAAGCGGGTGCCCTC
GTGCTTGCGGACAAGGGGATTGCGGCTGTCGACGAGCTTGACAAGATGCGGTGTGTGACTGGCGACTCCCTCGTCCACCT
CGCTGACGGGGGAATCAAACGCATCAGAGACCTTGCACACGACGCTGCATGTGAGGGGTCTATCGAGGAATTACCGAACG
GTCGAACGATTCGGAACGTCGACGCAGAAGCGTGGACGATGACTGAGGACGGGAGATTGGTGACTCGACCGGTCACGGCT
ATCCATGAATACGATGCACCGGATGAACTGACACAGATAACGTTGCAGTCCGGTGAGCAACTCACCGCGATTCACGACCA
CCCGTTCATCACGTTCGAAAACGGTGAGCGTGTAGAGAAGCCCGCAGCAGATGTCTCCCCCGGCGATTGGGTCTACGTCC
CGTGTCACCTCTCTCCCCGAGCGACGGATGGGGGCGTCCTAACTGCCGAATCGACCGCTGAGGCGACGATCACTGCCACC
GAAGACGAACTCTCCCCTGCGTTTGGTGCCATACTCGGCTACCTTTCCGGCGACGGGAACGTGTATTACAACCGTGAGGC
GGGCTCCTACGGTATCCGATTCACCAACAAGGAGGAGGAACTGCTCGCCGACTTCGAGCAGGTCTGTCGCGACACCTTCG
GTGTGGAACCGGTTCGCCCCCGTCTGAACAGCGTGACGACGGCGTCGAGACCGTACGGCTACCGGNCCGTGAGTACGCTG
ACGCGGTACTTTGATGCCGGGATGAACCTCGAATCTTATGATGGAAANCGTTCCNNNTGCCGTTTCGGAGGCCTTCGCTG
CGGCGAAGCGCGTTCGTGCNCGCGATGCTGGATAGCGAANNNGTGTCGATACCGATTCCGNACGTCAAANNNCNCTTTNN
NNNNNCGANTGCNNCTCGNNNTNCNNGAGANNNNCTCCTCTCTTNGGANTTTNCNNN

>Colony5_Reverse
GCGACGACGTATTCGGGTTTGCCGCGCCGAGGAGCGAACAACGCGATTTGAGCGTCGCGTTGATACCCGCCTTGGAGACC
GAAATCTGCTGTTGTTCGAGCGCTTCGTGCATAGCCGAACGGTCTTCGGAGTTGTGCACGACCATTCCATTGGCGACGAA
GTTGTGCGTTCCCTCGACAGTCAGGTCGTACACCTTTGGCTTCGAACGGGACCCGATGGCATCGAGAAGGCGTCGAGCAT
CCGAACGAATGTCGGAGAAGCGCGCGTTTGCCACCTCAACCACGTCAGTAAACCGGTCCGTCTCGACGACGCCGTTGAAC
CACCGCGAGACGGTCGAGCCAGCAACATCGAGGTCGCGCGCGATGTCAGCGAAAGAGACACCGTGGCGTTCGAGTTCGGA
ACGAAGTTCGCTCCAGTCGTCTGCACGCGGTTCGTGATCTAACAGCTGCTGTGCGTGTTCGACGGCCGCATCACCCTCGA
CGCCGAGGAGGTCCGCAAGCTCGTGTTTTAGTAGCCGAATTCGGTCGTCGGCATCGCTCTCGTCGACTGTTTCGACAGCC
TTCACGCGTCTCCACTTGACATCACCCCGAACGAGTCGTCGGATCGATTCGAGGTCCGTGTCAGCGACTGATTCAACGAT
TTCACGGAGCCGGCCTCTCGTGACTGCTCTGAGTTCGTCGTCCGTACCCCAGAGCCGAGAGATCTGTTGCTGGCTGAACG
AAGTTCCCACTGCCAACTCAGACTGAGAGACGTGGTAACGTTCTTTAACAATCGAAATCGTCGCCCAGTCACACGGTTCA
GAGAGTTGCGAGACGTCTGACTCGGCCTGTGACATTCGACTCTCAAACGCGTCGAGAACCGTTCGAGCACATGAGAACGA
GATGTTCGCGTCCTCGTTCTCGAAGTTGCAGTAGGTCGAATCGACGAGTCCGCATTCGAGACGTGAAGCCGTAGCGACTC
GCGNGCGACGATAGTACCCCCTCCACAGTTCGGANCACGTCGAGATCGTTCGNCACCCGAGACGACTCACAGACCTCGTC
AAGCGCCGATTGTTTGCGTTCCAGTGTGATTCCNATNTGNCNNNNCNNCNNNNNNGTCTGCTGCTNNNCCGCNAGTACGT
NNAGNNCNNNNNGANNNNNCGCGGTGCTGAATNTGCNNCNNNNNGCCCGNNN

>Colony6_Forward
CANNCGNCGCGTCCGANCCGCCGAAAATGGGTCTACGACCTCGAAGTCGAAGGCACCCACAGCTACGTTTCCAACGGGCT
CGTCTCGCATAATTCTCAGATGCTGTCATATATTCGAAATATCGCGCCGCGTTCTGTCTACACCTCCGGGAAGGGTTCTT
CGTCAGCAGGGCTCACCGCTGCGGCGGTTCGCGACGATTTCGGTGACGGCCAGCAGTGGACGCTCGAAGCGGGTGCCCTC
GTGCTTGCGGACAAGGGGATTGCGGCTGTCGACGAGCTTGACAAGATGCGGTGTGTGACTGGCGACTCCCTCGTCCACCT
CGCTGACGGGGGAATCAAACGCATCAGAGACCTTGCACACGACGCTGCATGTGAGGGGTCTATCGAGGAATTACCGAACG
GTCGAACGATTCGGAACGTCGACGCAGAAGCGTGGACGATGACTGAGGACGGGAGATTGGTGACTCGACCGGTCACGGCT
ATCCATGAATACGATGCACCGGATGAACTGACACAGATAACGTTGCAGTCCGGTGAGCAACTCACCGCGATTCACGACCA
CCCGTTCATCACGTTCGAAAACGGTGAGCGTGTAGAGAAGCCCGCAGCAGATGTCTCCCCCGGCGATTGGGTCTACGTCC
CGTGTCACCTCTCTCCCCGAGCGACGGATGGGGGCGTCCTAACTGCCGAATCGACCGCTGAGGCGACGATCACTGCCACC
GAAGACGAACTCTCCCCTGCGTTTGGTGCCATACTCGGCTACCTTTCCGGCGACGGGAACGTGTATTACAACCGTGAGGC
GGGCTCCTACGGTATCCGATTCACCAACAAGGAGGAGGAACTGCTCGCCGACTTCGAGCAGGTCTGTCGCGACACCTTCG
GTGTGGAACCGGTTCGCCCCCCGTCTGAACAGCGTGACGACGGCGTCGAGACCGTACGGCTACCGGGCCGTGAGTACGCT
GACGCGTACTTGATGCCGGGNTGAACCTCGAAATCTATGATGGAAAAGCGTTCCCGNTGCGTTTCGGAGGCTTCCNCTGG
CNNCGAAAGCGNCGTTCGTGCCGCGCGATGNTGAATAGCNNAGGNGTGTCGGATNNNNATCGNNACGTCAATNAGNTNNT
CNNNTACCGANNGNNGTCGGNNNCAAGGAGGNNNCCTCNTCTTGGAGTTCGGNCGNNNNNNA

>Colony6_Reverse
GCGACGACGTATTCGGGTTTGCCGCGCCGAggAGCGAACAACGCGATTTGAGCGTCGCGTTGATACCCGCCTTGGAGACC
GAAATCTGCTGTTGTTCGAGCGCTTCGTGCATAGCCGAACGGTCTTCGGAGTTGTGCACGACCATTCCATTGGCGACGAA
GTTGTGCGTTCCCTCGACAGTCAGGTCGTACACCTTTGGCTTCGAACGGGACCCGATGGCATCGAGAAGGCGTCGAGCAT
CCGAACGAATGTCGGAGAAGCGCGCGTTTGCCACCTCAACCACGTCAGTAAACCGGTCCGTCTCGACGACGCCGTTGAAC

```
CACCGCGAGACGGTCGAGCCAGCAACATCGAGGTCGCGCGCGATGTCAGCGAAAGAGACACCGTGGCGTTCGAGTTCGGA
ACGAAGTTCGCTCCAGTCGTCTGCACGCGGTTCGTGATCTAACAGCTGCTGTGCGTGTTCGACGGCCGCATCACCCTCGA
CGCCGAGGAGGTCCGCAAGCTCGTGTTTTAGTAGCCGAATTCGGTCGTCGGCATCGCTCTCGTCGACTGTTTCGACAGCC
TTCACGCGTCTCCACTTGACATCACCCCGAACGAGTCGTCGGATCGATTCGAGGTCCGTGTCAGCGACTGATTCAACGAT
TTCACGGAGCCGGCCTCTCGTGACTGCTCTGAGTTCGTCGTCCGTACCCCAGAGCCGAGAGATCTGTTGCTGGCTGAACG
AAGTTCCCACTGCCAACTCAGACTGAGAGACGTGGTAACGTTCTTTAACAATCGAAATCGTCGCCCAGTCACACGGTTCA
GAGAGTTGCGAGACGTCTGACTCGGCCCTGTGACATTCGACTCTCAAACGCGTCGAGAACCGTTCGAGCACATGAGACGA
GATGTTCGCGTCCTCGTTCTCGAAGTTGCAGTAGGTCGAATCGACGAGTCCGCATTCGGAGACGTGAAGCCGTAGCGACT
CGCGNGCGACGATAGTACCCNNCACNAGTTCGAATCACGTCGAGAATCGTTCGGTCACCCGAGACGNNTCACAGACCTCG
TCAGCGCCGATTGTTGCGTTCAGTGTGATCCGATATGCNNGAACCNNGNGGAGTCTGCTGCTNTNACCGCAGTACGTACA
GNTCCGTNNNNNNTNNGGNNNNCTTGANCTTGNCNNCNGGAACCCNCNAAANNNNCCNNN
```

Fig. S9: Sanger sequences from mext-a and d after invasion.

Table S1. Strains used in this study.

Strain	Genotype	Source / Reference
WR646	*Hfx. mediterranei* ATCC 33500*: ΔpyrE2*, *ΔtrpA*	Turgeman-Grott, 2019(2)
Vmcm a+d (UG743)	ΔpHV2, *ΔpyrE2, ΔhdrB*, intein a and intein d inserted into the *mcm helicase. p.S401P.*	This study
Vmcm a (UG654)	ΔpHV2, *ΔpyrE2, ΔhdrB*, intein a inserted into the *mcm helicase*	This study

Table S2. Plasmids used in this study.

Plasmid	Description	Source / Reference
pTA230	pBluescript II containing *Hfx. volcanii*'s pHV2 replication origin and *pyrE2 gene* under control of the ferredoxin promoter	Allers et al., 2004 (3)
mext-a (UG615)	pTA230 with 2155 bp of *Hfx. mediterranei mcm* gene: intein a extein and over 1000bp flanking sequences from each side. Gene block ordered from IDT and cloned using Gibson assembly	This study
mext-d (UG614)	pTA230 with 2089 bp of *Hfx. mediterranei mcm* gene: intein d extein and over 800bp flanking sequences from each side. Gene block ordered from IDT and cloned using Gibson assembly	This study
mext-a short (UG505)	pTA230 with 35 bp exteins sequence for intein a from *Hfx. mediterranei mcm* gene. Inserted through Gibson assembly in the MCS region of the plasmid	This study
mext-d short (UG639)	pTA230 with 103 bp exteins sequence for intein d from *Hfx. mediterranei mcm* gene cloned with HindIII and XbaI	This study
vext-a short (UG511)	pTA230 with 35 bp exteins sequence for intein a from *Hfx. volcanii mcm* gene. Inserted through Gibson assembly in the MCS region of the plasmid	This study
vext-d short (UG638)	pTA230 with 103 bp exteins sequence for intein d from *Hfx. volcanii mcm* gene cloned with HindIII and XbaI	This study
med-a,d target plasmid (UG488)	pTA230 with 2109 bp of *Hfx. mediterranei mcm* gene without intein a or intein d. cloned cloned with HindIII and BamHI and overlapping PCR	This study
pTA1478	Based on pTA230 with over 6 KB from *Hfx. volcanii* genome including the *mcm helicase* gene	Kindly provided by Prof. Thorsten Allers

Table S3. Oligonucleotides used in this study.

Primer	Sequence (5'-3')	Properties
IS667	CGCGCGTAATACGACTCACTA	Used to build mext-a and mext-d plasmids. Amplify the insert. Forward
IS668	CACTAAAGGGAACAAAAGCTGGA	Used to build mext-a and mext-d plasmids. Amplify the insert. Reverse
IS669	CCCGGGGGATCCACTAGTTC	Used to build mext-a and mext-d plasmids. Amplify the pTA230 plasmid. Forward
IS670	CTTATCGATACCGTCGACCTC	Used to build mext-a and mext-d plasmids. Amplify the pTA230 plasmid. Reverse
IS470	GACCCTGGTACAGGGAAGTCTCAGATGCTGTCATAAT ATCGAATTCCTGCAGCCC	Used to build mext-a short plasmid. Forward
IS471	TATGACAGCATCTGAGACTTCCCTGTACCAGGGTCCA AGCTTATCGATACCGTCGAC	Used to build mext-a short plasmid. Reverse
IS487	GACCCCGGAACTGGCAAATCGCAGATGTTATCATAAT ATCGAATTCCTGCAGCCC	Used to build vext-a short plasmid. Forward
IS488	TATGATAACATCTGCGATTTGCCAGTTCCGGGGTCCA AGCTTATCGATACCGTCGAC	Used to build vext-a short plasmid. Reverse
IS367	AAGCTTGGATTGCGGCTGTCGACGAGC	Used to build mext-d short plasmid. Forward with HindIII site
IS368	TCTAGAATACCCGCCTTGGAGACCG	Used to build mext-d short plasmid. Reverse with XbaI site
IS369	AAGCTTGTATCGCGGCGGTCGACGAAC	Used to build vext-d short plasmid. Forward with HindIII site
IS370	TCTAGAATGCCGGCCTTGGAGACCGA	Used to build vext-d short plasmid. Reverse with XbaI site
IS437	AAAAGGATCCTCGAGACCTTACAGAGCGGT	Used to build med-a,d target plasmid. Up forward: BamHI restriction site
IS438	TCTGAGACTTCCCTGTACCAGGGTCCCCTATCAGAAG CATGT	Used to build med-a,d target plasmid. Up reverse
IS439	TGGTACAGGGAAGTCTCAGATGCTGTCATATATTCGAA ATATCGCGC	Used to build med-a,d target plasmid. Mid forward
IS440	AGCCGAACGGTCTTCGGACCGCATCTTGTCAAGCTCG TCG	Used to build med-a,d target plasmid. Mid reverse
IS441	ACGAGCTTGACAAGATGCGGTCCGAAGACCGTTCGG CTAT	Used to build med-a,d target plasmid. Down forward
IS442	AAAAAAGCTTGCATAGTGCGGATGAGTTCG	Used to build med-a,d target plasmid. Down reverse: HindIII restriction site
IS498	GCCTCTTCGCTATTACGCCA	Used to amplify MCS cloning region on pTA230. Product size 292bp. Forward primer
IS499	CAAGCGCGCAATTAACCCTC	Used to amplify MCS cloning region on pTA230. Product size 292bp. Reverse primer
IS542	GTCAAAGGGCGAAAAACCGT	Used to screen for intein "a" integration into mext-a plasmid. Forward primer

IS543	GGGTCGACGAACTCTGAACC	Used to screen for intein "a" integration into mext-a plasmid. Reverse primer
IS687	ACATCGGATGGGAGCGAATC	Used to screen for intein "b" integration into mext-b plasmid. Forward primer
IS688	TCGCCGATGGATTCGTACTG	Used to screen for intein "b" integration into mext-b plasmid. Reverse primer
IS461	TGCTGACGAAGAACCCTTCC	Used to screen for intein "a" integration into med-a,d target plasmid. Forward primer
IS462	TGTGTGGAATTGTGAGCGGA	Used to screen for intein "a" integration into med-a,d target plasmid. Reverse primer
IS459	GGCGAATTGGGTACCGGG	Used to screen for intein "b" integration into med-a,d target plasmid. Forward primer
IS460	GGAAGGGTTCTTCGTCAGCA	Used to screen for intein "b" integration into med-a,d target plasmid. Reverse primer
GM25	CCAACGTCAAAGGGCGAAAA	Used to screen for intein "a" integration into pTA1478 target plasmid. Forward primer
GM26	ATGCTTCCGGCTCGTATGTT	Used to screen for intein "a" integration into pTA1478 target plasmid. Reverse primer

Dataset S1 (separate file). Multiple sequence alignment of 129 MCM protein sequences from Haloferacales with inteins and exteins in distinct partitions in nexus file format. Opened in seaview (4), the file retains the distinct sequence partitions.

Dataset S2 (separate file). MCM a, MCM d and MCM extein phylogenies in Newick format. Opened in seaview (4) of figtree (5) the file retains branch lengths and support values for each of the three trees.

SI References

1. L. T. Nguyen, H. A. Schmidt, A. von Haeseler, B. Q. Minh, IQ-TREE: a fast and effective stochastic algorithm for estimating maximum-likelihood phylogenies. *Mol Biol Evol* **32**, 268–274 (2015).

2. I. Turgeman-Grott, *et al.*, Pervasive acquisition of CRISPR memory driven by inter-species mating of archaea can limit gene transfer and influence speciation. *Nat Microbiol* **4**, 177–186 (2019).

3. T. Allers, H. P. Ngo, M. Mevarech, R. G. Lloyd, Development of additional selectable markers for the halophilic archaeon Haloferax volcanii based on the leuB and trpA genes. *Appl Environ Microbiol* **70**, 943–953 (2004).

4. M. Gouy, E. Tannier, N. Comte, D. P. Parsons, Seaview Version 5: A Multiplatform Software for Multiple Sequence Alignment, Molecular Phylogenetic Analyses, and Tree Reconciliation. *Methods Mol Biol* **2231**, 241–260 (2021).

5. A. Rambaut, FigTree v. 1.4.4. *http://tree.bio.ed.ac.uk/software/figtree/* (2018).
